# Conjugation of Native-Like HIV-1 Envelope Trimers onto Liposomes Using EDC/Sulfo-NHS Chemistry: Requirements and Limitations

**DOI:** 10.3390/pharmaceutics12100979

**Published:** 2020-10-16

**Authors:** Ehsan Suleiman, Julia Mayer, Elisabeth Lehner, Bianca Kohlhauser, Alexandra Katholnig, Mirjam Batzoni, Dominik Damm, Vladimir Temchura, Andreas Wagner, Klaus Überla, Karola Vorauer-Uhl

**Affiliations:** 1 Polymun Scientific Immunbiologische Forschung GmbH, 3400 Klosterneuburg, Austria; andreas.wagner@polymun.com; 2 Department of Biotechnology, University of Natural Resources and Life Sciences, 1190 Vienna, Austria; julia.mayer.97@students.boku.ac.at (J.M.); elehner@students.boku.ac.at (E.L.); biancak1708@gmail.com (B.K.); alex.alex.alex.alex@live.at (A.K.); miri.batzoni@googlemail.com (M.B.); karola.vorauer-uhl@boku.ac.at (K.V.-U.); 3 University of Vienna, 1010 Vienna, Austria; 4 FH Campus Wien, University of Applied Sciences, 1100 Vienna, Austria; 5 Institute of Clinical and Molecular Virology, Universitätsklinikum Erlangen, 91054 Erlangen, Germany; dominik.damm@uk-erlangen.de (D.D.); vladimir.temchura@fau.de (V.T.); klaus.ueberla@fau.de (K.Ü.)

**Keywords:** covalent conjugation, EDC/Sulfo-NHS, HIV-1, intrastructural help, liposomes, native-like Env trimers, particulate display, pre-concentration, protein-liposome conjugates, tag-free conjugation, vaccines

## Abstract

The display of native-like human immunodeficiency virus type 1 envelope (HIV-1 Env) trimers on liposomes has gained wide attention over the last few years. Currently, available methods have enabled the preparation of Env-liposome conjugates of unprecedented quality. However, these protocols require the Env trimer to be tagged and/or to carry a specific functional group. For this reason, we have investigated *N*-(3-Dimethylaminopropyl)-*N*′-ethylcarbodiimide/*N*-Hydroxysulfosuccinimide (EDC/Sulfo-NHS) chemistry for its potential to covalently conjugate tag-free, non-functionalized native-like Env trimers onto the surface of carboxyl-functionalized liposomes. The preservation of the liposome’s physical integrity and the immunogen’s conformation required a fine-tuned two-step approach based on the controlled use of β-mercaptoethanol. The display of Env trimers was strictly limited to activated liposomes of positive charge, i.e., liposomes with a positive zeta potential that carry amine-reactive Sulfo-NHS esters on their surface. In agreement with that, conjugation was found to be highly ionic strength- and pH-dependent. Overall, we have identified electrostatic pre-concentration (i.e., close proximity between negatively charged Env trimers and positively charged liposomes established through electrostatic attraction) to be crucial for conjugation reactions to proceed. The present study highlights the requirements and limitations of potentially scalable EDC/Sulfo-NHS-based approaches and represents a solid basis for further research into the controlled conjugation of tag-free, non-functionalized native-like Env trimers on the surface of liposomes, and other nanoparticles.

## 1. Introduction

The advent of native-like Env trimers has substantially contributed to the ongoing efforts to develop prophylactic vaccines against HIV-1 [1]. Despite the introduction of these well-conceived immunogen constructs and the use of new and elaborate immunisation strategies, inducing the desired type of immune response remains a major challenge. The reasons for this are multifaceted and have recently been discussed elsewhere [2,3]. The nanoparticulate display of native-like Env trimers and other state-of-the-art HIV immunogens has long been regarded as a promising strategy to improve and shape immune responses [4,5,6,7,8,9,10]. Liposomes, in particular, have gained wide attention over the last years. However, recent studies have dampened the expectations with regard to the potential of liposomal display to significantly improve the immune response against the envelope protein of HIV-1 [2,7,8].

The preparation of Env-liposome conjugates has been repeatedly published and is, with one exception, based on the high-affinity complexation of polyhistidine-tagged Env trimers (His-tagged Env trimers) with nitrilotriacetic acid-functionalised liposomes (NTA-functionalised liposomes) [11,12,13,14,15,16,17,18,19,20]. Bale et al. have reported the preparation of similarly packed liposomes using Env trimers with C-terminal cysteines and maleimide-functionalised liposomes [16]. Several groups have also described the preparation of conjugates using double-functionalised liposomes (i.e., liposomes that include both NTA-functionalised lipids and maleimide-functionalised lipids) in order to overcome the issue of poor serum stability of non-covalent Env-liposome conjugates [12,16,18]. These preparation techniques have all yielded liposomes of unprecedented quality with high numbers of uniformly oriented Env trimers on their surface. Moreover, the underlying chemistry is well-described and easy to implement. Conjugation protocols for both tag-based (i.e., non-covalent conjugation onto NTA-functionalised liposomes) and tag-assisted (i.e., covalent conjugation onto double-functionalised liposomes) proceed with high conjugation efficiency at around 100% (unpublished observations). They come, however, with some limitations regarding the stability of the conjugates they yield, their scalability, and their application for the production of clinical-grade Env-liposome conjugates. Non-covalent conjugates (in particular those that use NTA-functionalised lipids such as 1,2-dioleoyl-sn-glycero-3-[(*N*-(5-amino-1-carboxypentyl) iminodiacetic acid) succinyl] (nickel salt) (18:1 DGS-NTA(Ni))) readily dissociate in serum and are as such not suitable for any in vivo application or investigations under physiological conditions [16,18]. Although there is at least one approved protein-based therapeutic that comprises a His-tag (Blinatumomab), the use of conjugation techniques that rely on the use of a His-tag, might raise concerns and impede regulatory approval, especially as it has been repeatedly reported that Env-liposome conjugates prepared this way induce an anti-His immune response [11]. With regard to their stability under physiological conditions, covalent Env-liposome conjugates are clearly superior [16,18]. All protocols for the preparation of covalent Env-liposome conjugates published so far involve the use of maleimide-functionalised liposomes [12,16,18]. Maleimides, however, are known to hydrolyse in a pH-, temperature- and time-dependent manner, which impedes a straightforward upscaling of these protocols [21,22]. Small-scale preparations of Env-liposome conjugates are easily performed within a couple of hours and, within this context, the loss of maleimide activity may have simply been counteracted by using disproportionate molar fractions of maleimide-functionalised lipids in the liposomal membrane [16,18]. For large-scale preparations where the production of the maleimide-functionalised liposomes and the conjugation itself have to be performed on different days (or at least with a considerably longer waiting time than for small-scale preparations) maleimide hydrolysis represents a serious complication. The longer waiting times arise from the fact that the requirements with regard to in-process controls and accurately defined process conditions are different in a pharmaceutical industry setting [23].

Given the above-mentioned hurdles and the fact that all protocols published so far require the native-like Env trimers to carry a terminal tag or a specific functional group, we have decided to explore frugal conjugation approaches applicable to completely tag-free, non-functionalised Env trimers. ConSOSL.UFO.664 (UFO Env), an uncleaved pre-fusion optimised construct was used as a model native-like Env trimer [24,25]. It is an extensively studied state-of-the-art HIV immunogen (with a well-described antigenic and immunogenic profile) that is currently under clinical investigation [24].

The use of EDC/Sulfo-NHS is well-characterised and mediates the formation of a covalent bond between primary amines and carboxyl groups, i.e., an amide bond [26,27,28]. It is a widely used zero-length crosslinker and reactions are described as proceeding with high efficiency. With this in mind, we have decided to systematically investigate it for its potential to covalently couple native-like Env trimers on the surface of carboxyl-functionalised liposomes. EDC/Sulfo-NHS-based conjugation to carboxyl-functionalised nanoparticles has been described for many other proteins and peptides, but was only recently described for the first time within the context of the particulate display of native-like Env trimers [29,30,31,32].

We reasoned that we were unlikely to obtain the degree of controlled, oriented conjugation that can be achieved by using site-directed conjugation or the methods published by others dealing with the preparation of Env-liposome conjugates. We therefore decided that conjugation to a flexible linker might be more beneficial as it would render relevant epitopes (but admittedly also the potentially immunodistractive base) more accessible. For this purpose, we have used liposomes that contain lipids that carry their carboxyl group at the distal end of a short monodisperse polyethylene glycol chain, i.e., 1,2-distearoyl-sn-glycero-3-phosphoethanolamine-*N*-[carboxy(polyethylene glycol)] with 14 polyethylene glycol units (DSPE-PEG14-COOH).

Published experimental work on the conjugation of proteins on functionalised surfaces and the interaction of proteins with lipid membranes suggest that appropriate surface concentrations of protein are a prerequisite for conjugation reactions to proceed efficiently [33,34,35,36,37,38,39,40,41,42]. This is particularly relevant for conjugation reactions performed at low(er) molar bulk concentrations of protein. Generally, appropriate surface concentrations can be achieved through pre-concentration or (to some extent) by increasing the bulk concentration of the protein during conjugation. Adequate pre-concentration can, for example, be reached through high-affinity complexation (e.g., His-tag/NTA) or through electrostatic attraction. For this reason, we decided to comprehensively explore the role of electrostatic pre-concentration within the context of an EDC/Sulfo-NHS-based approach. We have investigated the effect of liposome charge, ionic strength, and pH on conjugation efficiency and the conformational integrity of the liposome-displayed Env trimers. Since this research was carried out within the context of the development of processes for the manufacturing of T helper liposomes (i.e., liposomes capable of harnessing intrastructural help that carry T helper cell peptides within their aqueous interior or lipid membrane and additionally display native-like Env trimers on their surface) special emphasis was placed on investigating parameters that may affect both the efficiency of helper peptide encapsulation and the efficiency of immunogen conjugation [43,44]. To the best of our knowledge, this is the first report dealing with the basic requirements of coupling tag-free, non-functionalised native-like HIV-1 Env trimers onto the surface of (helper peptide-loaded) liposomes. Overall, we are confident that the present study provides a solid basis for us and others to continue working on this and similar potentially scalable, tag-free approaches for the manufacturing of Env-liposome conjugates.

## 2. Materials and Methods

### 2.1. Materials

1,2-dioleoyl-sn-glycero-3-phosphocholine (DOPC) and 1,2-dioleoyl-sn-glycero-3-phospho-(1′-rac-glycerol) (sodium salt) (DOPG) were obtained from Lipoid GmbH, Ludwigshafen am Rhein, Germany. (3β)-cholest-5-en-3-ol (cholesterol) was obtained from Dishman Netherlands B.V., Veenendaal, Netherlands. 1,2-dioleoyl-3-trimethylammonium-propane (chloride salt) (DOTAP) was obtained from Merck KGaA, Darmstadt, Germany. 1,2-dioleoyl-sn-glycero-3-[(*N*-(5-amino-1-carboxypentyl) iminodiacetic acid) succinyl] (nickel salt) (18:1 DGS-NTA(Ni)) was obtained from Avanti Polar Lipids, Inc., Alabaster, AL, USA. Monodisperse 1,2-distearoyl-sn-glycero-3-phosphoethanolamine-*N*-[carboxy(polyethylene glycol)] with 14 polyethylene glycol units (DSPE-PEG14-COOH) was custom-synthesised with a purity of ≥95% (Biochempeg Scientific Inc., Watertown, MA, USA). All other lipids were of ≥98% purity. Methanol, chloroform, and ethanol used in the preparation of liposomes were of Reag. Ph. Eur. quality and were obtained from Merck KGaA, Darmstadt, Germany. *N*-Hydroxysulfosuccinimide sodium salt (Sulfo-NHS; ≥98%) and *N*-(3-Dimethylaminopropyl)-*N*′-ethylcarbodiimide hydrochloride (EDC; ≥99.0%) were also purchased from Merck KGaA, Darmstadt, Germany. The T helper cell peptide OVA 323–339 was custom-synthesised as TFA salt with a purity of ≥98% (piCHEM, Grambach, Austria). The tag-free, native-like HIV-1 envelope trimers ConM SOSIP.v7 (SOSIP Env), ConSOSL.UFO.664 (UFO Env) and their EDC-crosslinked, PGT145-purified versions were of clinical grade and were produced in-house (Polymun Scientific GmbH, Klosterneuburg, Austria) [24,45,46]. Antibodies 2G12, b12, PG9, and PGT145 were produced in-house (Polymun Scientific GmbH, Klosterneuburg, Austria) [47,48,49,50]. PGT121 was kindly provided by Lukas Mach and was obtained through the NIH AIDS Reagent Program [47]. The antibody ACS202 was a kind gift from Marit van Gils and Rogier W. Sanders [51]. 17b, 19b, PGT151, and VRC01 were provided by Rebecca Moore and Quentin Sattentau [52,53,54,55]. All chemicals used in the sample preparation and to prepare buffers and mobile phases were of analytical grade or of Ph. Eur. quality and were purchased from Merck KGaA, Darmstadt, Germany. The purified water (18.2 MΩ at 25 °C) used in this study was provided by an Ultra Clear UV unit (SG Wasseraufbereitung und Regenerierstation GmbH, Barsbüttel, Germany).

### 2.2. Buffers

Buffers were generally prepared as stock solution (2-fold), filtered through a 0.22 µm filter and stored at −20 °C. Tables S1–S4 summarise the composition of all buffers used in this study.

### 2.3. Liposome Preparation

#### 2.3.1. Liposome Composition

Liposomes used in the optimisation of the conjugation reaction were composed of 4 mol% DSPE-PEG14-COOH, x mol% DOTAP or DOPG (x = 0, 3, 6, 9, 12 or 15) and (96 − x) mol% DOPC. In general, if not stated otherwise, liposomes were composed of 4 mol% DSPE-PEG14-COOH, 15 mol% DOTAP 81 mol% DOPC. Liposomes that were part of the investigation into the effect of liposome charge on protein conjugation were prepared by thin-film hydration. All other liposomes were prepared by cross-flow injection as described below.

#### 2.3.2. Thin-Film Hydration

Liposomes were prepared by thin-film hydration as described in Suleiman et al., 2019, with minor modifications [44]. Briefly, lipids were dissolved in chloroform/methanol (2:1, by volume) and transferred into a round-bottomed flask. The solvent was evaporated by applying a stepwise increasing vacuum: 2 min at atmospheric pressure, 5 min at 450 mbar and 15 min at <40 mbar. The evacuation was performed under constant rotation of 150 rpm and at a temperature of 40 °C. The thin lipid film formed as result of this procedure was flush-dried under a stream of nitrogen. The lipid film was hydrated with buffer and peptide solution to prepare empty liposome and peptide-loaded liposomes, respectively. Hydrations for the preparation of cationic peptide-loaded liposomes (i.e., loaded with the model T helper cell peptide OVA 323–339) were performed with a low-ionic-strength buffer with a pH above the peptide’s isoelectric point of 6.5 (e.g., 5 mM BB sucrose pH 8.5 *w*/300 mM sucrose) whereas hydrations for the preparation of anionic peptide-loaded liposomes were performed at pH values well below 6.5, e.g., with 5 mM AB sucrose pH 4.0 *w*/300 mM sucrose [56,57,58]. After hydration, liposomes were stepwise downsized at 40 °C with a LIPEX™ Extruder (Northern Lipids Inc., Burnaby, BC, Canada) as described in one of our previous publications [44].

#### 2.3.3. Cross-Flow Injection

Larger quantities of liposomes (up to 200 mL preparation volume) were prepared using a scalable cross-flow injection setup [59,60]. Briefly, an ethanolic lipid solution was mixed with buffer (with or without T helper cell peptide) using a proprietary mixing module. The preparation was performed in a heating cabinet at up to 60 °C. Downsizing by means of extrusion was performed at room temperature on an EmulsiFlex-C5 (Avestin Inc., Ottawa, ON, Canada) equipped with 100 nm Whatman^®^ Nuclepore track-etched membranes (GE Healthcare UK Limited, Buckinghamshire, UK).

#### 2.3.4. Purification by Tangential Flow Filtration (TFF)

TFF was used for the removal of ethanol and non-encapsulated (i.e., free and outer surface-associated) peptides as described elsewhere [44]. Briefly, 100 kDa mPES MicroKros^®^ modules (Repligen, Waltham, MA, USA) with a surface area of 20 cm^2^ (for small-scale preparations with a production volume below 10 mL) or 115 m^2^ (for larger preparations with a production volume of up to 100 mL) were used. Up to 10 cycles of filtration (volume exchanges) using a buffer with a high ionic strength (e.g., 5 mM PBS pH 6.5 *w*/150 mM NaCl) were performed. Subsequently, additional 10 filtration cycles were performed using a reaction buffer, i.e., a buffer in which the activation and conjugation reactions described below take place. In some cases, liposomes were also concentrated to obtain lipid concentrations required for subsequent conjugation reactions. Liposomes were then sterile-filtered using appropriate syringe filters and stored at 2–8 °C.

### 2.4. Particle Size and Zeta Potential

#### 2.4.1. General

Z-average diameter, polydispersity index (*PdI*), and zeta potential were determined in triplicate on a Zetasizer Nano ZS (Malvern Instruments Ltd., Malvern, UK). Measurements were performed at 25 °C in disposable folded capillary cells (DTS1070). Samples were diluted to a total lipid concentration in the range of 50 µM to 500 µM with an appropriate buffer, i.e., the same buffer in which they were prepared or in which the activation/conjugation was performed. Properties of the dilution buffer relevant to the calculation of the particle size or the zeta potential, were calculated using the complex solvent builder add-in (Zetasizer Nano ZS software, version 7.03).

#### 2.4.2. Titrations

Titrations with 0.1 M HCl were performed using an MPT-2 autotitrator (Malvern Instruments Ltd., Malvern, UK) in order to determine the zeta potential of liposomes or native-like envelope trimers as a function of pH. Liposomes were diluted with a low-ionic-strength buffer (5 mM BB sucrose pH 8.5 *w*/300 mM sucrose) to a lipid concentration of 250 µM, whereas native-like HIV-1 envelope trimers were diluted to a concentration of 83 nM (30 µg/mL).

### 2.5. Quantification of Peptides and Lipids by HPLC

Peptides and lipids were quantified using sample preparation techniques and RP-HPLC methods described in detail in Suleiman et al., 2019 [44]. Total lipid concentrations were extrapolated from DOTAP concentrations.

### 2.6. Peptide Recovery and Peptide Encapsulation Efficiency

Peptide recoveries (i.e., the fraction of T helper cell peptides that remains encapsulated after particular process steps) (PR [%]) were calculated on the basis of the peptide concentration of the liposomes before activation ($\beta_{A,\;Peptide}$) and the peptide concentration after the conjugation reaction has been quenched and after non-encapsulated peptides have been completely removed as described below ($\beta_{B,\;Peptide}$) (Equation (1)). The peptide encapsulation efficiencies (i.e., the amount of encapsulated T helper cell peptide in relation to the amount of peptide initially used) (EE [%]) were calculated on the basis of the peptide concentration after extrusion ($\beta_{X,\;Peptide}$) and the peptide concentration after the complete removal of non-encapsulated peptides by means of TFF ($\beta_{Y,\;Peptide}$) (Equation (2)).

Processing-related losses (i.e., losses of peptides and lipids associated with tangential flow filtration, sterile filtration, etc.) were corrected for by considering the lipid concentrations at relevant reaction steps ($\beta_{A,\;Lipid};\beta_{B,\;Lipid};\;\beta_{X,\;Lipid};\;\beta_{Y,\;Lipid}$).

(1) $$PR\;\left[\%\right]=\frac{\beta_{B,\;\;Peptide\;\cdot }\;\beta_{A,\;\;Lipid}}{\beta_{A,\;\;Peptide\;\cdot }\;\beta_{B,\;\;Lipid\;}}\cdot 100$$

(2) $$EE\;\left[\%\right]=\frac{\beta_{Y,\;Peptide\;\cdot }\;\beta_{X,\;\;Lipid}}{\beta_{X,\;\;Peptide\;\cdot }\;\beta_{Y,\;\;Lipid\;}}\cdot 100$$

### 2.7. Protein Bioconjugation

#### 2.7.1. General

**Figure 1:**
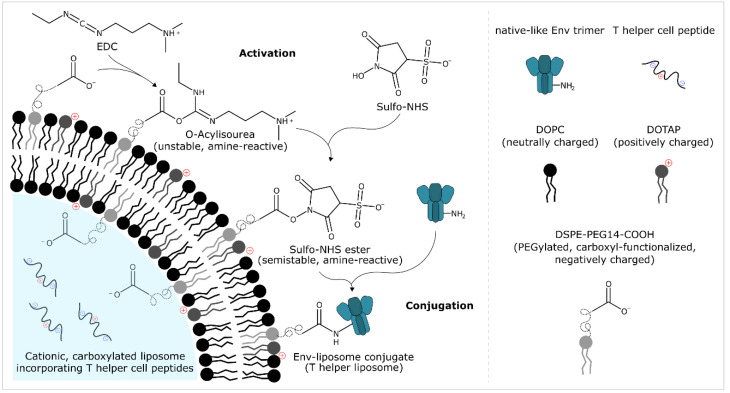
EDC/Sulfo-NHS-based preparation of Env-liposome conjugates (T helper liposomes): conjugation of native-like HIV-1 envelope trimers onto helper peptide-loaded liposomes. Carboxyl-functionalised liposomes are activated with EDC/Sulfo-NHS. Excess activation reagents are then removed by filtration-based separation techniques or through chemical inactivation using β-mercaptoethanol (not shown). Native-like Env trimers are then added and covalent Env-liposome conjugates are formed. Env-liposome conjugates that additionally incorporate T helper cell peptides (derived from commonly used licensed protein vaccines) are referred to as T helper liposomes. These liposomes are designed to harness intrastructural help, i.e., to recruit pre-existing, non-cognate T helper cells upon administration in order to provide help to Env-reactive/Env specific B cells and eventually shape/improve the immune response against the liposome-displayed Env trimers [43]. First steps towards the development of liposome-based vaccines capable of harnessing intrastructural help date back to the early 1990′s [61,62,63]. Recent studies that involved particle-based vaccines (virus-like particles and calcium phosphate nanoparticles) have provided additional proof of concept for this type of strategy within the context of HIV-1 [64,65,66,67,68]. Recent independent reports on liposome-based vaccines (against HIV-1, malaria, group A streptococci and ErbB-2 overexpressing breast cancer) utilizing the same principle are also very encouraging [69,70,71,72].

The covalent conjugation of tag-free, native-like HIV-1 envelope trimers onto carboxyl-functionalised liposomes was performed using an EDC/Sulfo-NHS-based approach. The protocol comprises four steps: activation, removal of excess activation reagents by gel filtration or through chemical inactivation, conjugation, and stopping the conjugation reaction. A detailed description of these four steps is given below. Small-scale reactions (≤1.5 mL total reaction volume) were performed in regular polypropylene tubes in an Eppendorf ThermoMixer^®^ at 1400 rpm. Reactions with higher reaction volumes were performed in borosilicate glass bottles under constant stirring. The specific reaction conditions of the individual experiments performed as part of this study (e.g., the molar excess of reagents, the buffers used for particular reaction steps, the reaction time, the molar Env:funct. lipid ratio, etc.) are given in full detail in the figure captions of the respective experiments. Figure 1 shows a reaction scheme of the main chemical reactions, i.e., activation and conjugation.

#### 2.7.2. Activation

A 4-fold to 40-fold molar excess of Sulfo-NHS and a 10-fold to 100-fold molar excess of EDC over the accessible carboxyl groups on the outer surface of the liposomes were used for activation, i.e., for the formation of an amine-reactive Sulfo-NHS ester. In general, if not stated otherwise, a 4-fold molar excess of Sulfo-NHS and a 10-fold molar excess of EDC were used. The molar ratio of Sulfo-NHS to EDC was kept constant at 1:2.5 throughout all experiments. We assumed a random distribution of carboxyl-functionalised lipids across the internal and external leaflet of the liposome, i.e., we considered 50% of all carboxyl-functionalised lipids of the liposome to be available for activation and subsequent conjugation. Liposomes were diluted to have a total lipid concentration of 21.2 mM to 29.1 mM (which corresponds to a concentration of 424 µM to 582 µM accessible carboxyl-functionalised lipid) at activation. Both Sulfo-NHS and EDC were pre-weighed and stored at −20 °C in a desiccator. Aliquots were dissolved in water just before their use. First Sulfo-NHS and then EDC were added to the liposomes. Activations were performed in MES buffer (e.g., 50 mM MBS pH 6.1 or 50 mM MB sucrose pH 6.1) or 5 mM PB pH 6.0 *w*/15 mM NaCl and *w*/270 mM sucrose. The reaction was allowed to proceed for 15 min at room temperature.

#### 2.7.3. Removal/Inactivation of Excess Activation Reagents

Excess activation reagents were removed by one or two consecutive gel filtrations through NAP™-5 columns or PD-10 columns (GE Healthcare UK Limited, Buckinghamshire, UK). Liposomes were eluted with the desired conjugation buffer, e.g., 5 mM PB pH 6.0 *w*/15 mM NaCl and *w*/270 mM sucrose. Alternatively, excess EDC was chemically inactivated by the addition of a 1-fold to 20-fold molar excess β-mercaptoethanol over the amount of EDC used in the activation of the carboxyl-functionalised liposomes. The reaction was allowed to proceed for 1 to 20 min at room temperature. In general, if not stated otherwise, EDC was inactivated by the addition of a 1.75-fold molar excess β-mercaptoethanol. This quenching reaction was allowed to proceed for 3.75 min before initiating the conjugation reaction.

#### 2.7.4. Conjugation

The conjugation reaction is initiated within 0.5 to 2 min after the removal of excess activation reagents. For this purpose, tag-free, native-like HIV-1 envelope trimers (in appropriate buffer) are added to the activated liposomes to give a 31-fold to 252-fold molar excess of accessible carboxyl groups over the Env trimer’s three N-termini at conjugation. In general, if not stated otherwise, conjugation reactions were performed at a nominal total lipid concentration of 10 mM and an Env trimer concentration of 706 nM (254 µg/mL), which corresponds to a 94-fold molar excess of accessible carboxyl groups over the Env trimer’s three N-termini. As with all reaction steps described earlier, this is done under constant shaking/stirring. In some cases (e.g., in those reactions that were part of the investigation into the effect of pH on protein conjugation), the pH is adjusted by the addition of NaOH or HCl just before the initiation of the reaction. The conjugation reaction was then allowed to proceed for 2 to 6 h at 25 °C. The reaction was then stopped with a 10-fold molar excess of glycine over the accessible carboxyl groups on the liposomes.

### 2.8. Ultracentrifugation

A floatation-based ultracentrifugation setup was used to separate free (non-conjugated) Env trimers from liposomes and Env-liposome conjugates. This protocol enables the separation of non-encapsulated peptides as it is performed under electrostatic binding-impeding conditions described in one of our previous publications on the encapsulation of hydrophilic T helper cell peptides into liposomes [44]. The protocol was adapted from that of Tronchere and Boal, with several modifications [73]. A SORVALL Discovery M150 SE micro-ultracentrifuge equipped with a 12-Place S45-A fixed-angle rotor (Thermo Fisher Scientific, Waltham, MA, USA) was used for this purpose. The ultracentrifugation was performed in regular polypropylene tubes. First, a 300 µL sample (referred to as T (for total conjugation reaction mix) as it is the sample collected after stopping the conjugation reaction and it contains non-conjugated Env trimers, liposomes as well as Env-liposome conjugates) was mixed with 300 µL of an aqueous NaCl/sucrose solution to give a final NaCl and sucrose concentration of 0.15 M and 1.0 M, respectively. Next, 450 µL of an aqueous solution with 0.15 M NaCl and 0.7 M sucrose were carefully overlaid. Finally, 150 µL of an aqueous solution with 0.15 M NaCl and 0.3 M sucrose were overlaid. The gradient was then centrifuged at 99,000× *g* for 1 h, at 4 °C. Following this, 900 µL of the solution were removed from the bottom of the tube using a Hamilton syringe. This fraction is referred to as NCF 1 (non-conjugate fraction 1) as it mainly contains free Env trimers and is collected after the first cycle. The remaining 300 µL are referred to as ICF (intermediate conjugate fraction) as they predominantly contain the floated liposomes/Env-liposome conjugates with some carryovers, i.e., residual non-conjugated Env trimers. A second ultracentrifugation with the same gradient is performed with the 300 µL of ICF in order to remove these residual non-conjugated Env trimers. The 900 µL removed from the bottom of that solution are referred to as NCF 2 (non-conjugate fraction 2) while the remaining 300 µL are referred to as CF (conjugate fraction). NCF 1 and NCF 2 were each concentrated to a volume of 300 µL using Amicon^®^ Ultra-0.5 mL centrifugal filters with a cut-off of 30 kDa (Merck KGaA, Darmstadt, Germany).

### 2.9. Sodium Dodecyl Sulfate–Polyacrylamide Gel Electrophoresis (SDS-PAGE)

T, CF, NCF 1 and NCF 2 were loaded on reducing SDS-PAGE to estimate the extent of conjugation and the degree of intramolecular EDC-crosslinking of the Env trimers. Reducing SDS-PAGE were performed on a XCell SureLock™ Mini-Cell Electrophoresis System using Invitrogen™ NuPAGE™ protein gels (Thermo Fisher Scientific, Waltham, MA, USA) according to the manufacturer’s instructions. Samples were run on 4–12% Bis-Tris gels. Gels were stained with Imperial™ Protein Stain (Thermo Fisher Scientific, Waltham, MA, USA) using a two-hour staining protocol.

### 2.10. Enzyme-Linked Immunosorbent Assay (ELISA): Quantitative Analyses and Antigenicity Analyses

Nunc MaxiSorp™ 96-well plates were coated overnight at 2–8 °C with 2G12 at 2.0 µg/mL. The coated plates were then washed four times with washing buffer (5 mM PBS pH 7.5 *w*/150 mM NaCl and *w*/0.1% Tween 20) using a plate washer. Plates were then blocked with dilution buffer (washing buffer *w*/1% bovine serum albumin (BSA) and *w*/1% Triton X-100) for 1 h at room temperature. Plates were then washed four times and the samples were added. Samples (T and CF) were diluted with the Triton X-100-containing dilution buffer. Calibrations in the range of approximately 10 ng/mL to 500 ng/mL were done with soluble, tag-free, native-like HIV-1 envelope trimers used in the conjugation experiments. For antigenicity analyses, samples (CF and soluble Env trimers) were diluted to a protein concentration of 500 ng/mL. For this purpose, samples were analysed with a 2G12-based ELISA, i.e., an ELISA that only detects 2G12-reactive Env trimers. Plates were then allowed to incubate for 1 h at room temperature. Plates were then washed, and biotinylated detection antibody was added. In the case of the quantitative 2G12-based ELISA, biotinylated 2G12 was added at a concentration of 5.0 µg/mL while in the case of the quantitative PGT145-based ELISA (i.e., an ELISA that only detects PGT145-reactive Env trimers), biotinylated PGT145 was added at a concentration of 1.0 µg/mL.

For antigenicity analyses, a 1:3 serial dilution of biotinylated antibodies was prepared to give an antibody concentration in the range of approximately 10 µg/mL to 5 ng/mL. After an incubation of 1 h at room temperature, plates were washed four times. Streptavidine-POD conjugate (1:5000) was then added and plates were allowed to incubate for 25 min at room temperature before they were washed eight times. A solution of o-phenylenediamine (OPD) and hydrogen peroxide in citric acid buffer was added to initiate the chromogenic reaction. The reaction was stopped with sulphuric acid. Absorption was measured at 492 nm (reference wavelength: 630 nm).

### 2.11. Antibody Biotinylation

Antigenicity analyses were performed with a panel of monoclonal anti-HIV-1 antibodies that target a range of different important sites of the envelope protein, i.e., the CD4 binding site (VRC01 and b12), the N160 glycan-dependent site associated with the V1/V2 loops (PGT145 and PG9), the N332 glycan-dependent site at the base of the V3 loop (PGT121), CD4-induced epitopes (17b), the gp120-gp41 interface (PGT151 and ACS202), the V3 loop (19b) and the high-mannose patch centred around the glycan at N322 (2G12). For this purpose, antibodies were biotinylated with EZ-Link™ Sulfo-NHS-LC-Biotin (Thermo Fisher Scientific, Waltham, MA, USA) according to the manufacturer’s instructions with the only modification that reactions were performed at antibody concentrations of 1 mg/mL. Excess reagents were removed by washing three times with 5 mM PBS pH 6.5 *w*/150 mM NaCl using Amicon^®^ Ultra-0.5 mL centrifugal filters with a cut-off of 30 kDa (Merck KGaA, Darmstadt, Germany). Antibodies were sterile-filtered and stored at −20 °C or 2–8 °C until further analysis/use. Antibody concentrations were determined using a NanoDrop^®^ ND-1000 UV-Vis Spectrophotometer (Thermo Fisher Scientific, Waltham, MA, USA). The sample type was set to IgG.

### 2.12. Conjugation Efficiency

The conjugation efficiency (CE) was calculated on the basis of the protein concentration of the total conjugation reaction mix [$\beta_{T,\;Env\;\left(2G12\right)}$] and that of the conjugate fraction [$\beta_{CF,\;Env\;\left(2G12\right)}$] (Equation (3)). The lipid concentrations of both fractions [$\beta_{T,\;Lipid\;\left(2G12\right)};\beta_{CF,\;Lipid\;\left(2G12\right)}$] were considered to correct for ultracentrifugation-related losses. Protein concentrations were determined using a 2G12-based ELISA.

(3) $$CE\;\left[\%\right]=\frac{\beta_{CF,\;Env\;\left(2G12\right)\;\cdot }\;\beta_{T,\;Lipid\;\left(2G12\right)}}{\beta_{T,\;Env\;\left(2G12\right)\;\cdot }\;\beta_{CF,\;Lipid\;\left(2G12\right)}}\cdot 100$$

### 2.13. PGT145 Reactivity

The PGT145 reactivity ($R_{PGT145}$) is calculated on the basis of the protein concentrations of the conjugate fraction as determined by PGT145-based ELISA [$\beta_{CF,\;Env\;\left(PGT145\right)}$] and 2G12-based ELISA [$\beta_{CF,\;Env\;\left(2G12\right)}$] (Equation (4)).

(4) $$R_{PGT145\;}\left[\%\right]=\frac{\beta_{CF,\;Env\;\left(PGT145\right)}}{\beta_{CF,\;Env\;\left(2G12\right)}}\cdot 100$$

## 3. Results and Discussion

### 3.1. Efficient Removal of Residual EDC Improves the PGT145 Reactivity of Liposome-Displayed Env Trimers

EDC/Sulfo-NHS-based approaches for the preparation of bioconjugates are well-established but may come with limitations with regard to their scalability. This is because the amine-reactive Sulfo-NHS ester formed as result of the activation is known to be readily hydrolysable and unstable (Figure 1). Furthermore, excess EDC has to be removed before initiation of the conjugation reaction as it will otherwise induce intramolecular cross-linking of the Env trimers. Although chemical cross-linking within the context HIV-1 immunogen design is seen as a promising approach for stabilisation and immune focusing, it represents an undesired side reaction in EDC/Sulfo-NHS-based approaches for the particulate display of native-like HIV-1 envelope trimers [46,74]. The main reason for this is that chemical cross-linking is a stochastic process that gives rise to a heterogenous population of trimers in different conformational states. Chemical cross-linking usually requires subsequent purification (e.g., affinity chromatography using trimer-specific PGT145 antibodies) in order to selectively enrich envelope trimers of desired conformational state; this method is not applicable within the context of the preparation of Env-liposome conjugates.

Initial screening studies performed in order to identify appropriate reaction conditions showed that the Env trimer’s native-like structure is highly affected by residual EDC present during the conjugation step. The intensity of trimer bands on the reducing SDS-PAGE increased with increasing molar excess of EDC used for activation (Figure S1A). The appearance of these trimer bands indicates the formation of covalent cross-links within the trimeric envelope protein and suggest a gradual loss in the protein’s native-like conformation. Similarly, binding to the trimer- and apex-specific antibody PGT145 (i.e., PGT145 reactivity) decreased with increasing concentrations of EDC (Figure S1B). In contrast, the conjugation efficiency increased and plateaued around 100% at a 25-fold molar excess of EDC over the accessible carboxyl groups on the outer surface of the liposomes (Figure S1B). The amount of Env trimers per total lipid was determined to be in the range of 0.04 µmol/mmol which corresponds to approximately three to four Env trimers per liposome (Figures S1B and S2A) [19]. The low number of Env trimers per liposome is a consequence of the comparatively low Env trimer:liposome ratio selected for our investigations into tag-free conjugation via EDC/Sulfo-NHS chemistry. Thus, a conjugation efficiency of 100% generally corresponds to roughly four to five Env trimers per liposome (Figure S2B). Importantly, control experiments with non-activated liposomes show that the fraction of electrostatically bound Env trimer is negligible (Figure S1). The gradual loss in PGT145 reactivity is explained by the fact that a single gel-filtration step (as performed in this early set of screening experiments) only achieves a limited degree of buffer-exchange and thus incomplete removal of excess EDC. According to the manufacturer’s documentation, desalting capacities are in the range of >90% to >98% and depend on the protocol (i.e., on whether a spin or gravity protocol is used) and the type of the gel-filtration column. Taken together, these initial experiments suggest keeping the molar excess of EDC for activation as low as possible and/or to perform two consecutive gel-filtration steps in order to keep the concentrations of residual EDC low.

### 3.2. The Poor Aqueous Stability of Sulfo-NHS Esters Impedes the Scalability of EDC/Sulfo-NHS-Based Approaches for the Preparation of Env-Liposome Conjugates

**Figure 2:**
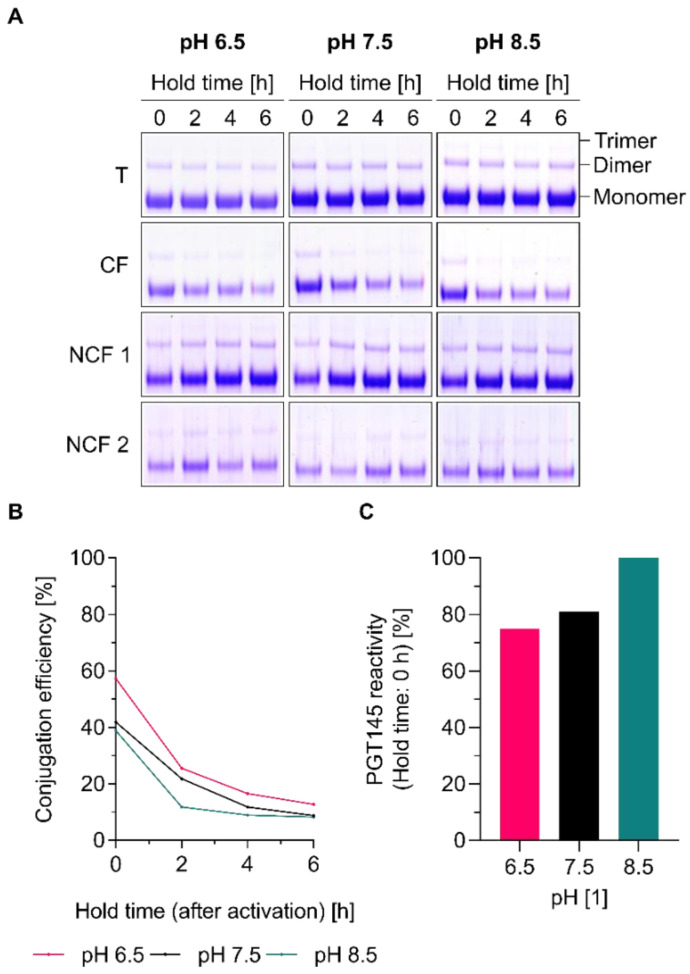
Conjugation efficiency and PGT145 reactivity as a function of pH and the apparent reactivity of the amine-reactive intermediate (Sulfo-NHS ester). (**A**) Reducing SDS-PAGE showing the extent of covalent conjugation onto cationic liposomes. (**B**,**C**). (**B**) Conjugation efficiency and (**C**) PGT145 reactivity after a hold time of 0 h as determined by ELISA. Data (ELISA) represent the mean from two analytical replicates. (**A**–**C**). Liposomes were activated in 50 mM MB sucrose pH 6.1. Excess activation reagents were removed by two consecutive gel-filtration steps. Liposomes were eluted with 5 mM PB (*w*/15 mM NaCl and *w*/270 mM sucrose) with a pH of 6.5, 7.5, and 8.5, respectively. Conjugation was initiated by the addition of UFO Env after 0, 2, 4, and 6 h, respectively. The panel shows the results from a single explorative screening experiment. Abbreviations: T, total conjugation reaction mix; CF, conjugate fraction; NCF 1, non-conjugate fraction 1; NCF 2, non-conjugate fraction 2. See Section 2.8 for further information.

Since this research is done within the context of the development of readily scalable approaches for the manufacturing of Env-liposome conjugates, we have performed several experiments to explore alternatives to gel filtration, which is only applicable for small-scale preparations. To this end, we have performed experiments to assess the stability of the amine-reactive Sulfo-NHS ester and to estimate the available time (e.g., for hypothetical processing steps such as the removal of excess activation reagents by tangential flow filtration) between activation and the initiation of conjugation. Experiments were conceived so as to determine conjugation efficiency as a function of hold time (i.e., the time that has passed between activation and the initiation of conjugation) and pH (Figure 2). Different pH conditions were included in our experiment as it is known that both conjugation and the hydrolyses of Sulfo-NHS esters are pH-dependent [26]. For this purpose, liposomes were activated, gel filtrated twice (in order to efficiently remove excess activation reagents and investigate the impact of different pH conditions) and stored for 0, 2, 4 or 6 h before initiating conjugation by the addition of UFO Env trimers. The initiation of the conjugation directly after activation and gel filtration (i.e., after a hold time of 0 h) resulted in conjugation efficiencies in the range of approximately 40% to 60% (Figure 2B). Conjugation efficiencies across all tested pH conditions as estimated by SDS-PAGE and ELISA are, however, substantially reduced after a hold time of 2 h and approach values below 15% after a hold time of 6 h (Figure 2A,B). PGT145 reactivity is largely maintained as judged by the results of the ELISA and the absence of cross-linked trimers on the reducing SDS-PAGE (Figure 2A,C). In agreement with the literature on EDC/Sulfo-NHS chemistry, the active intermediate’s stability (as indirectly assessed by the extent of conjugation as a function of the hold time) is reduced with increasing pH [26].

While tangential flow filtration represents an excellent and readily scalable method to purify protein-liposome conjugates (i.e., to efficiently remove non-conjugated protein, byproducts/side products and unreacted starting materials at the end of a conjugation reaction), our experiments indicate that it is not suitable for the purification of activated liposomes. Altogether, the results confirm our initial reservations with regard to the scalability of EDC/Sulfo-NHS-based conjugation approaches that would rely on time-consuming filtration-based separation techniques for the removal of excess EDC.

### 3.3. Chemical Inactivation of Residual EDC by β-Mercaptoethanol Is a Time-Saving and Potentially Scalable Alternative to the Removal by Filtration-Based Methods

**Figure 3:**
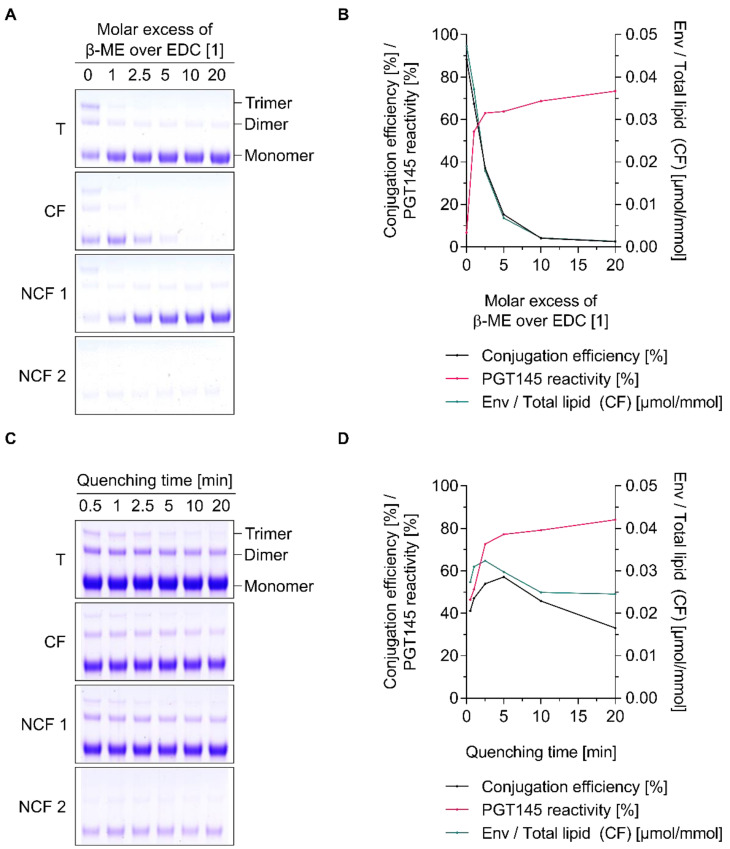
Concentration- and time-dependent quenching of excess EDC by β-mercaptoethanol (β-ME). (**A**,**C**) Reducing SDS-PAGE showing the extent of covalent conjugation of UFO Env trimers onto cationic liposomes as a function of (**A**) the used molar excess of β-ME and (**C**) the quenching time (i.e., the time β-ME was allowed to react with EDC before initiating the conjugation by the addition of UFO Env), respectively. (**B**,**D**) Conjugation efficiency, PGT145 reactivity and the amount of conjugated UFO Env per lipid as a function of (**B**) the used molar excess of β-mercaptoethanol and (**D**) the quenching time, respectively. (**A**–**D**) Liposomes were activated in 5 mM PB pH 6.0 *w*/15 mM NaCl and *w*/270 mM sucrose. (**A**,**B**) Excess activation reagents were deactivated by the addition of a 0-, 1-, 2.5-, 5-, 10- and 20-fold molar excess β-ME over the amount of EDC used for activation. Conjugation was initiated by the addition of UFO Env after a quenching time of 10 min. (**C**,**D**) Excess activation reagents were deactivated by the addition of a 1.75-fold molar excess β-ME over the amount of EDC used for activation. Conjugation was initiated by the addition of UFO Env after a quenching time of 0.5, 1, 2.5, 5, 10, and 20 min, respectively. Data (ELISA and lipid quantification) represent the mean from two analytical replicates. The panel shows the results from a single explorative screening experiment. Abbreviations: T, total conjugation reaction mix; CF, conjugate fraction; NCF 1, non-conjugate fraction 1; NCF 2, non-conjugate fraction 2. See Section 2.8 for further information.

As a result of this and as it has been widely employed by others, we have decided to look into the controlled use of β-mercaptoethanol as a method to chemically inactivate (i.e., quench) excess EDC (Figure 3) [26]. In the absence of β-mercaptoethanol and without prior removal of excess EDC reagents by gel filtration, conjugation reactions are found to be highly efficient with conjugation efficiencies around 90% (Figure 3A,B). As expected, the main fraction of liposome-associated Env trimers lost its trimeric conformation as indicated by the PGT145 reactivity of ≤10% and the presence of prominent trimer bands on the reducing SDS-PAGE (Figure 3A,B). Chemical inactivation of excess EDC using β-mercaptoethanol resulted in a concentration-dependent preservation of the PGT145 reactivity upon conjugation, but this was at the expense of the conjugation efficiency. A molar excess of β-mercaptoethanol over EDC as little as 1-fold resulted in a PGT145 reactivity and conjugation efficiency of approximately 55% and 65%, respectively (Figure 3A,B). The marked drop in conjugation efficiency is most easily explained by the reaction between the Sulfo-NHS ester (which is not just amine-reactive but nucleophile-reactive) and the thiol group of β-mercaptoethanol [26,28]. As part of the development of an appropriate quenching protocol for our purpose, we have also looked into the optimal duration of the quenching reaction, i.e., the time β-ME was allowed to react with EDC before initiating the conjugation by the addition of UFO Env (Figure 3C,D). At a constant 1.75-fold molar excess of β-mercaptoethanol over EDC, we have found the optimal quenching time (as judged by the conjugation efficiencies of around 50% and PGT145 reactivities of ≥70%) to be in the range of 2.5 min to 10.0 min (Figure 3C,D). In comparison, conjugation experiments in which residual EDC was removed by gel filtration resulted in conjugation efficiencies in the range of 40% to 60% and PGT145 reactivities in the range of 70% to 100% (Figure 2). These experiments show that β-mercaptoethanol is effective in inactivating excess EDC. Chemical inactivation is time-saving, largely preserves the Env trimer’s delicate conformation and may, in theory, be easily scaled up. Taken together, our experiments demonstrate that the controlled use of β-mercaptoethanol is a viable alternative to filtration-based separation techniques.

### 3.4. The Charge of Both Liposomes and Env Trimers Determines the Extent of Conjugation

In order to explore the role of liposome charge, peptide-loaded, carboxyl-functionalised liposomes with increasing molar fractions of DOPG or DOTAP were prepared and characterised (Figure S3). As expected, the zeta potential decreased with increasing molar fraction of DOPG but increased with increasing molar fractions of DOTAP (Figure S3B). Particle diameter and *PdI* were unaffected by the addition of DOPG at around 90 nm and 0.10, respectively (Figure S3A). In contrast, particle diameter and *PdI* slightly increased with increasing molar fractions of DOTAP. Here, particle diameter and *PdI* were in the range of approximately 95 nm to 120 nm and 0.10 to 0.20, respectively. In agreement with recently published data, the encapsulation of the hydrophilic, non-conformational model T helper cell peptide OVA 323–339 was dependent on the presence of charged lipids (i.e., DOPG or DOTAP) in the liposome formulation [44]. Encapsulation efficiencies increased with increasing molar fractions of charged lipid (Figure S3D).

**Figure 4:**
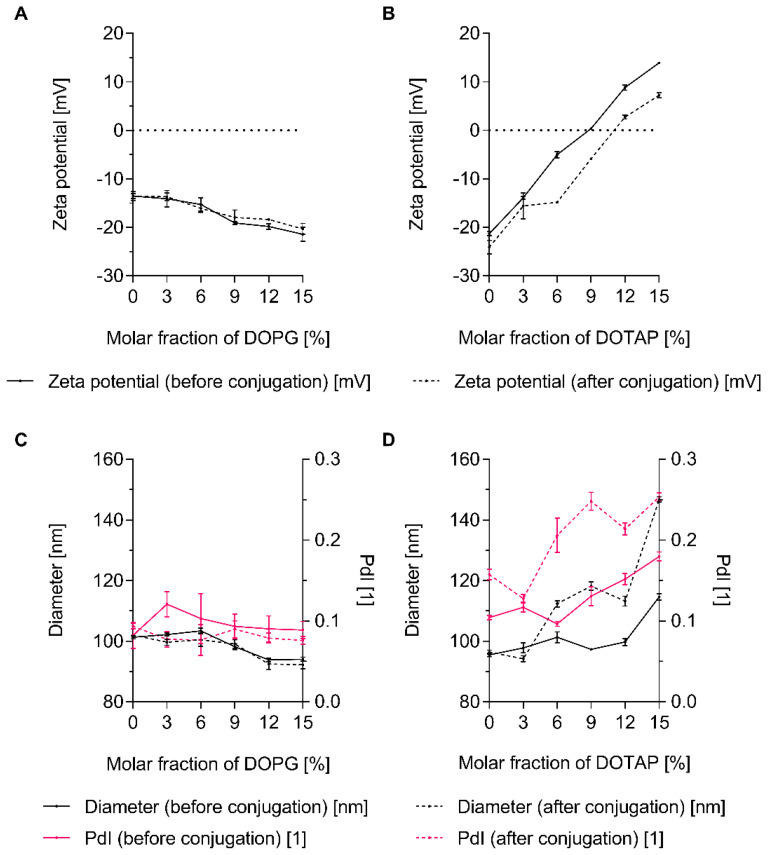
Liposome charge-dependent conjugation of UFO Env trimers: physico-chemical characterisation of liposomes before and after conjugation. (**A**,**B**) Zeta potential of carboxyl-functionalised liposomes with increasing fractions of (**A**) DOPG and (**B**) DOTAP. (**C**,**D**) Diameter, (i.e., Z-average diameter) and *PdI* of carboxyl-functionalised liposomes with increasing fractions of (**C**) DOPG and (**D**) DOTAP. Data represent the mean ± standard deviation from three analytical replicates. The panel shows the results from a single explorative screening experiment.

**Figure 5:**
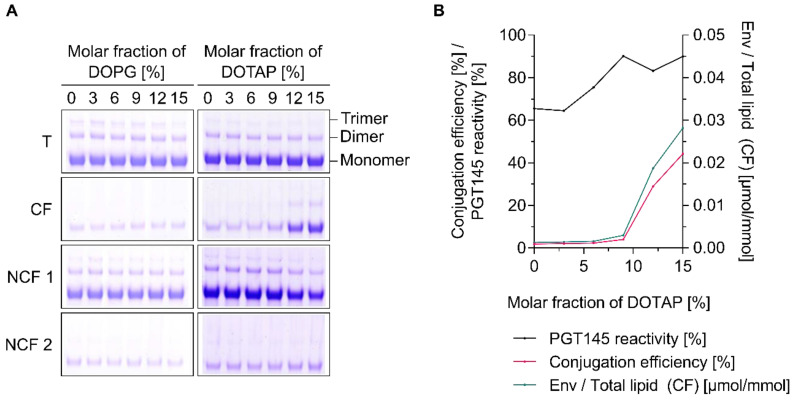
Liposome charge-dependent conjugation of UFO Env trimers. (**A**) Reducing SDS-PAGE showing the extent of covalent conjugation onto liposomes as a function of increasing molar fractions of DOPG (left panel) or DOTAP (right panel) in liposomal membranes. (**B**) Conjugation efficiency, PGT145 reactivity and the amount of conjugated UFO Env per lipid. Data (ELISA and lipid quantification) represent the mean from two analytical replicates. (**A**,**B**) Liposomes of the DOPG series were activated in 50 mM MB sucrose pH 6.1. Excess activation reagents were removed by gel filtration. Liposomes were eluted with 5 mM PBS pH 6.5 *w*/150 mM NaCl. In contrast, liposomes of the DOTAP series were activated in 5 mM PB pH 6.0 *w*/15 mM NaCl and *w*/270 mM sucrose. Excess EDC was chemically deactivated by the addition of β-ME. The panel shows the results from a single explorative screening experiment. **Abbreviations:** T, total conjugation reaction mix; CF, conjugate fraction; NCF 1, non-conjugate fraction 1; NCF 2, non-conjugate fraction 2. See Section 2.8 for further information.

Titrations under low-ionic-strength conditions (≤2 mS/cm) showed that the UFO Env trimer used in this set of experiments has an isoelectric point of approximately 4.3 which is in close agreement with reports on the dynamic electrophoretic fingerprinting of a range of HIV-1 envelope constructs (Figure S4A) [75]. UFO Env trimers thus exhibit a negative zeta potential over the entire pH range relevant to EDC/Sulfo-NHS-based conjugation techniques [26]. In accordance with this, neutrally or negatively charged liposomes did not engage in the formation of conjugates while liposomes exhibiting a positive zeta potential (i.e., those with molar fractions of DOTAP ≥12 mol%) did so readily (Figure 4 and Figure 5). The conjugation efficiency was zeta potential-dependent, while the PGT145 reactivity of the surface-displayed Env trimers did not appear to be affected by increasing zeta potentials, i.e., increasing molar fractions of DOTAP (Figure 4B and Figure 5B).

For Env-liposome conjugates incorporating 15 mol% DOTAP, the amount of Env trimers per total lipid was determined to be around 0.028 µmol/mmol, which corresponds to approximately two to three Env trimers per liposome (Figure 5B and Figure S2A). The amount of encapsulated T helper cell peptide per total lipid was approximately 100-fold higher at around 2.7 µmol/mmol (Figure S3D). Zeta potentials slightly decreased upon conjugation while, both particle diameter and *PdI* increased (Figure 4). After successful conjugation, particle diameter and *PdI* were in the range of 95 nm to 145 nm and 0.15 to 0.25, respectively.

In concordance with these findings, conjugation of a positively charged model protein (hen egg white lysozyme (HEL)) to cationic liposomes was not possible (data not shown). In contrast, covalent conjugation to negatively charged liposomes proceeded readily. All in all, these observations are in excellent agreement with published experimental work on the interaction of lysozymes and other proteins with liposomes [40,41,42]. Also in agreement with our observations, Pejawar-Gaddy et al. have reported that the inclusion of DOTAP to double-functionalised, DOPC-based interbilayer-cross-linked multilamellar vesicles (ICMVs) improved the conjugation efficiency of Env trimers from around 5% to slightly above 20% [17]. In stark contrast to our findings, Ringe et al. showed that Env trimers can be coupled to the surface of negatively charged nanoparticles using EDC/Sulfo-NHS chemistry [32]. The relatively high Env trimer:nanoparticle ratio and high Env trimer bulk concentration may have contributed to the higher number of displayed Env trimers. Moreover, one may speculate whether the introduction of positively charged, C-terminal, lysine-rich tags facilitated an electrostatic pre-concentration.

Studies on the interaction of serum proteins with nanoparticles have repeatedly shown that, at a given pH, proteins may also bind/interact irrespective of their net charge [76,77]. It has been reported that size, charge, charge density, nanoparticle composition and the type of functionalisation affect the composition of the protein corona. To the best of our knowledge, a clear, generalizable correlation between the net charge of the bound proteins and the charge of the nanoparticle, has never been established. It thus appears that phenomena and forces other than electrostatic attraction/repulsion play a significant role. Taken together, in contrast to what has been demonstrated in the context of the conjugation of Env trimers onto the surface of carboxy-functionalised iron oxide nanoparticles, our data indicate electrostatic pre-concentration to be an essential requirement for conjugation reactions to proceed. Moreover, our data show that the presence of charged lipids in the liposomal membrane affects both the conjugation efficiency as well as the efficiency with which hydrophilic T helper cell peptides are encapsulated. Importantly, a considerable number of T helper cell peptides relevant to the clinical translation of our liposome-based vaccine (e.g., those derived from tetanus toxoid or licensed vaccines against hepatitis B virus (HBV)) are less hydrophilic than the model peptide employed in the present study. In fact, many are hydrophobic and it is expected that their formulation is not or considerably less affected by the presence/absence of charged lipids [44,78].

### 3.5. The Extent of Conjugation Is a Function of Ionic Strength

**Figure 6:**
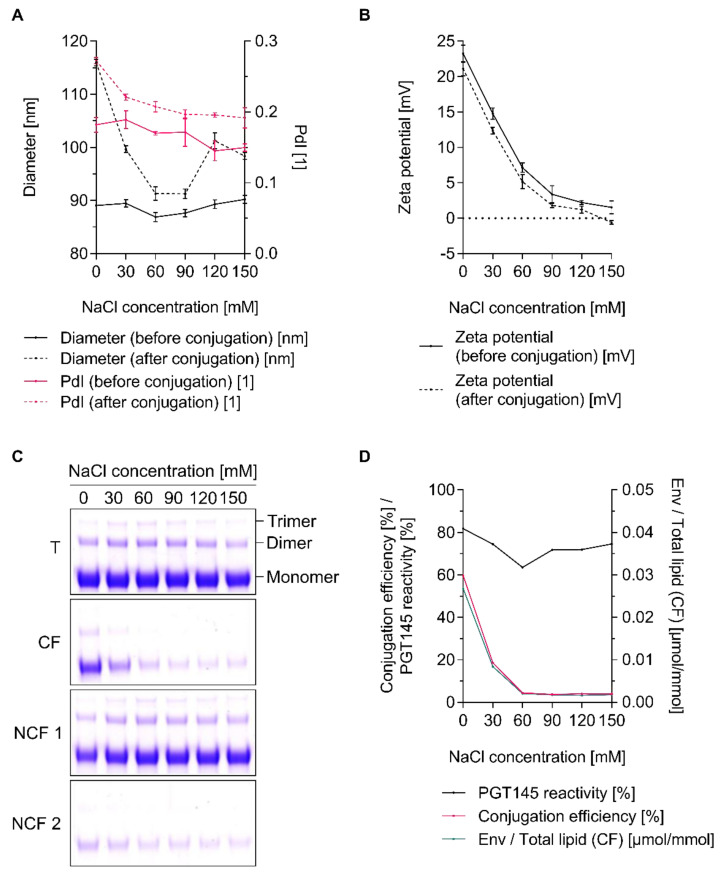
Ionic strength-dependent conjugation of UFO Env trimers onto cationic liposomes. (**A**,**B**) (**A**) Diameter, (i.e., Z-average diameter), *PdI* and (**B**) zeta potential of liposomes before and after conjugation. Measurements were performed with the corresponding conjugation buffers. Data represent the mean ± standard deviation from three analytical replicates. (**C**) Reducing SDS-PAGE showing the extent of covalent conjugation. (**D**) Conjugation efficiency, PGT145 reactivity and the amount of conjugated UFO Env per lipid. Data (ELISA and lipid quantification) represent the mean from two analytical replicates. (**A**–**D**) Conjugation experiments were performed in 5 mM PB pH 6.0 with a constant osmolality but with a varying ionic strength, i.e., varying concentrations of sodium chloride and sucrose. For this purpose, cationic liposomes dispersed in 5 mM PB sucrose pH 6.0 *w*/300 mM sucrose and liposomes of the same lipid composition but dispersed in 5 mM PBS pH 6.0 *w*/150 mM NaCl, were mixed to give the desired sodium chloride concentration. After activation of carboxyl groups, excess EDC was chemically deactivated by the addition of β-ME. The panel shows the results from a single explorative screening experiment. **Abbreviations:** T, total conjugation reaction mix; CF, conjugate fraction; NCF 1, non-conjugate fraction 1; NCF 2, non-conjugate fraction 2. See Section 2.8 for further information.

Preliminary experiments to identify appropriate reaction conditions for tag-free conjugation indicated the importance of ionic strength and pH for both conjugation efficiency and the physical integrity of the liposomes. For this reason, we have performed a comprehensive screening study (Figures S4B–D and S5). Particle diameter and *PdI* of cationic, carboxyl-functionalised liposomes, increased upon the addition of UFO Env trimers (Figure S5). The increase was pH- and ionic strength-dependent (i.e., sodium chloride concentration-dependent) with high pH and low ionic strength resulting in particles with higher particle diameter and *PdI*. Moreover, the onset appears to be dependent on whether the liposomes were activated with EDC/Sulfo-NHS or not. For non-activated liposomes the gradual increase was observed at a sodium chloride concentration ≤30 mM. In contrast, activated liposomes already showed an increase at a sodium chloride concentration ≤60 mM. Substantial agglomeration (i.e., Z-average diameter ≥ 150 nm and *PdI* ≥ 0.25) was only observed for sodium chloride concentrations ≤15 mM and pH values >7.5. As expected, the zeta potential gradually decreased with increasing sodium chloride concentration as a result of electric-field screening (Figure S4C,D) [44]. At pH 6.5 and a sodium chloride concentration of 0 mM, activated liposomes exhibited a zeta potential of approximately +20 mV, whilst at concentrations ≥60 mM zeta potentials were well below +5 mV. Independent of whether covalent conjugation occurred or not, zeta potentials decreased upon the addition of UFO Env trimers. In summary, our data indicate distinct contributions of charge screening by sodium chloride, activation by EDC/Sulfo-NHS and the addition of the anionic Env trimers, to the overall reduction in zeta potential. Importantly, notable conjugation of UFO Env trimers onto cationic liposomes was also only observed at lower ionic-strength-conditions, i.e., sodium chloride concentration ≤30 mM (Figure 6C,D). While the conjugation efficiency was clearly ionic strength-dependent, PGT145 reactivity was only negligibly affected by it (Figure 6D). It therefore appears that conjugation efficiency and the increase in particle size and *PdI*, go-hand-in hand. Generally, a slight increase in particle diameter and *PdI* as a result of a successful conjugation is expected and has been reported by others in the context of non-covalent, complexation-based conjugation [15]. Within the context of our tag-free conjugation approach, however, electrostatically driven agglomeration (i.e., ionic strength-dependent agglomeration) appears to represent an additional cause of this (apparent) increase in particle diameter. Clearly, agglomeration has major implications for the filterability and stability of the formed Env-liposome conjugates. Moreover, agglomeration is very likely to favour the leakage/loss of incorporated biomolecules, e.g., T helper cell peptides and adjuvants such CpG ODN and poly I:C. A sodium chloride concentration of 15 mM, however, turned out to be an acceptable compromise; it facilitates the required pre-concentration of UFO Env trimers and efficiently prevents agglomeration (Figure 6). Interestingly, varying the sodium chloride concentration within a range of 0 mM to 150 mM had no measurable effect on the extent of conjugation to negatively charged liposomes (data not shown). Moreover, it had no effect on the particle size of the liposomes. Investigations into whether increasing the ionic strength further could have reduced the electrostatic repulsion to a level that would have enabled a successful conjugation, were beyond the scope of this study. Taken together, these results demonstrate the significance of performing tag-free, pre-concentration-dependent conjugation at particular ionic-strength-conditions.

### 3.6. Conjugation Efficiency and PGT145 Reactivity of Liposome-Displayed Env Trimers Are a Function of pH

**Figure 7:**
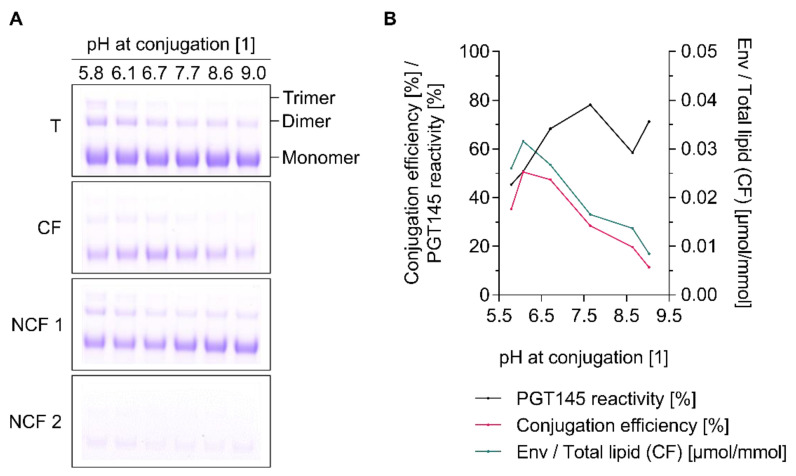
pH-dependent conjugation of UFO Env trimers onto cationic liposomes. (**A**) Reducing SDS-PAGE showing the extent of covalent conjugation. (**B**) Conjugation efficiency, PGT145 reactivity and the amount of conjugated UFO Env per lipid. Data (ELISA and lipid quantification) represent the mean from two analytical replicates. (**A**,**B**) Liposomes were activated in 5 mM PB pH 6.0 *w*/15 mM NaCl and *w*/270 mM sucrose. Excess EDC was chemically deactivated by the addition of β-ME. The desired pH was adjusted by adding 0.1 M NaOH or 0.1 M HCl right before initiation of the conjugation reaction. The indicated pH was measured at the end of the reaction, i.e., after stopping the reaction by the addition of glycine. Data on the physico-chemical properties of the liposomes before and after conjugation can be found in the supplement (Figure S6). The panel shows the results from a single explorative screening experiment. **Abbreviations:** T, total conjugation reaction mix; CF, conjugate fraction; NCF 1, non-conjugate fraction 1; NCF 2, non-conjugate fraction 2. See Section 2.8 for further information.

Next, a series of experiments was performed to investigate the role of pH in the tag-free conjugation of UFO Env trimers onto cationic, carboxyl-functionalised liposomes. Physico-chemical characterisation of the liposomes before and after conjugation indicate that the liposome’s integrity is preserved over the tested pH range (Figure S6). Both conjugation efficiency and the PGT145 reactivity of the surface-displayed Env trimers were found to be strongly pH-dependent (Figure 7). The optimal pH was found to be around 6.1. Reactions performed above or below pH 6.1 were found to be less efficient. The increased conjugation efficiency at slightly acidic pH values could be explained by the reduced hydrolysis rate of the Sulfo-NHS ester under these pH conditions. The absolute difference in the liposomes’ and the Env trimers’ zeta potential (i.e., |Δ ZP|) and thus the extent of electrostatic attraction peaks is the in the range of pH 5.5 to 6.5 (Figure S4A). In contrast, the optimal pH for maintaining the PGT145 reactivity of the UFO Env trimers was found to be around 7.7. Reactions performed above or below pH 7.7 resulted in the formation of Env-liposome conjugates with reduced PGT145 reactivity (Figure 7B). In agreement with this, the reducing SDS-PAGE showed prominent trimer bands with intensities increasing in a pH-dependent manner (Figure 7A).

Residual activation reagents may react with carboxyl groups present on the accessible surface of the Env trimers. Sulfo-NHS esters formed as a result of this react with primary amines in close proximity and form intramolecular peptide bonds. The increased formation of cross-linked trimers at lower pH values might be explained by the increased stability of these amine-reactive Sulfo-NHS esters. In contrast, the loss in PGT145 reactivity at higher pH values might be a consequence of the increased reactivity of the Env trimers’ primary amines, i.e., increased nucleophilicity as a result of the deprotonation at higher pH conditions [26]. Taken together, performing conjugation reactions in the pH range of 6.1 to 7.7 represents a reasonable compromise between achieving satisfactory conjugation efficiencies and preserving the immunogen’s conformational integrity.

### 3.7. Efficient Conjugation Is Limited to Particular Reaction Conditions

The number of His-tagged Env trimers that can be displayed on the surface of NTA-functionalised liposomes depends on a range of factors. These include the molar fraction of functionalised lipids in the liposomal membrane (such as the NTA-functionalised lipid and/or maleimide-functionalised lipid in the case of tag-assisted covalent coupling), the ratio of liposome to Env trimer at conjugation, and the lipid composition of the liposome. These have all been shown to be of importance [13,15,17,18]. A high density of Env trimers on the surface of liposomes is widely regarded as being critical to bring about the hypothesised benefits of particulate display of immunogens [4,5,7,8]. Recent studies on the role of nanoscale antigen organisation on the activation of B cells have, however, shed a new light on this much-debated aspect of molecular vaccine design [79].

We decided to explore the possibility of increasing both conjugation efficiency and the number of Env trimers per liposome without varying the lipid composition of the liposomes. Conjugation experiments in which the Env trimer:liposome ratios (i.e., the molar ratio of the N-termini of the trimeric envelope protein Env to the accessible carboxyl groups on the outer surface of the carboxyl-functionalised liposome) were varied over an extended range, showed that the number of Env trimers per liposome increased with increasing Env trimer:liposome ratio, although at a certain point (>1:72) this was at the expense of conjugation efficiency (Figure S7). Likewise, performing conjugation experiments at very low Env trimer:liposome ratios of <1:168 was accompanied by a loss in conjugation efficiency. Within this range, the conjugation efficiency was constant at approximately 40%. Experiments to improve the conjugation efficiency by increasing the bulk reactant concentration (but keeping the Env trimer:liposome ratio and other activation/conjugation parameters constant) indicate a total lipid concentration in the range of 2.4 mM to 12.1 mM (which corresponds to Env trimer concentrations in the range of 89 nM (32 µg/mL) to 567 nM (204 µg/mL) to be ideal for the cationic liposome formulation used in this study (Figure S8A). Further increasing the total reactant concentration was, however, shown to decrease the conjugation efficiency. Interestingly, the decrease in conjugation efficiency was accompanied by an increase in PGT145 reactivity. Taken together, these experiments show that efficient conjugation is restricted to particular reaction conditions.

### 3.8. Conjugation of Other Tag-Free, Non-Functionalised Next-Generation HIV-1 Immunogens

**Figure 8:**
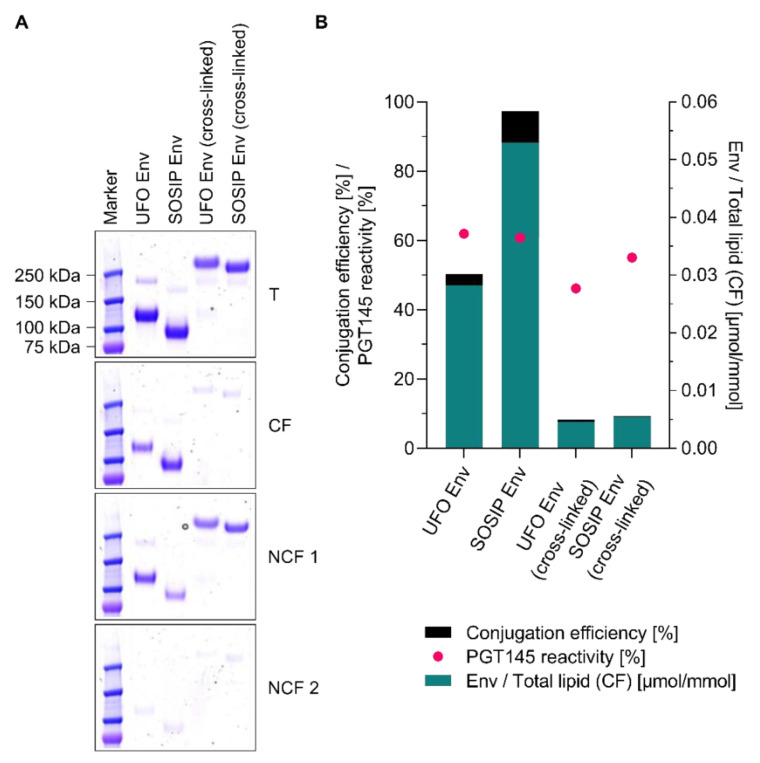
Conjugation of next-generation HIV-1 immunogens onto cationic, helper peptide-loaded liposomes. (**A**) Reducing SDS-PAGE showing the extent of covalent conjugation of UFO Env trimers, SOSIP Env trimers and their EDC cross-linked versions. Marker: Precision Plus Protein™ Kaleidoscope™ (Bio-Rad Laboratories, Inc., Hercules, CA, USA). (**B**) Conjugation efficiency, PGT145 reactivity and the amount of conjugated Env per lipid. Data (ELISA and lipid quantification) represent the mean from two analytical replicates. (**A**,**B**) Excess EDC was chemically deactivated by the addition of β-ME. The panel shows the results from a single explorative screening experiment. Data on the peptide recovery after conjugation as well as on the physico-chemical properties of the liposomes before and after conjugation can be found in the supplement (Figure S9). **Abbreviations:** T, total conjugation reaction mix; CF, conjugate fraction; NCF 1, non-conjugate fraction 1; NCF 2, non-conjugate fraction 2. See Section 2.8 for further information.

**Figure 9:**
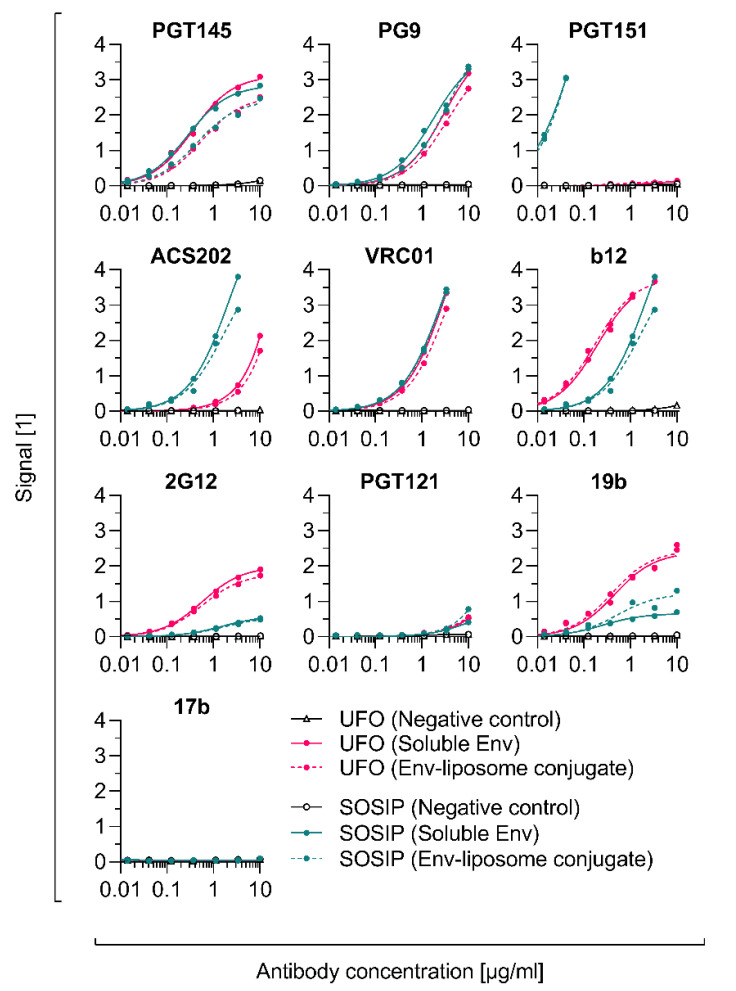
Antigenicity of soluble and liposome-displayed next-generation HIV-1 immunogens. Monoclonal antibody titrations of 2G12-captured Env or Env-liposome conjugates. The graphs show the binding curves of Triton X-100-treated *conjugate fractions* (CF), i.e., non-intact Env-liposome conjugates without non-conjugated Env. The concentration of the captured samples was determined using an 2G12-based ELISA and was the same (0.5 µg/mL) throughout all titrations. Data represent the mean from two analytical replicates.

In order to test whether tag-free conjugation onto cationic liposomes is also applicable to other clinically relevant state-of-the-art HIV-1 Env trimers, ConM SOSIP.v7 (SOSIP Env) was selected for further investigations [45]. Moreover, chemically stabilised (i.e., EDC-crosslinked) and PGT145-purified versions of UFO Env and SOSIP Env were selected for further conjugation experiments onto peptide-loaded liposomes [46,74]. Both UFO Env and SOSIP Env readily engaged in the formation of Env-liposome conjugates. Conjugation efficiencies and PGT145 reactivities were ≥50% and ≥60%, respectively (Figure 8). Titrations with a panel of conformational and non-conformational monoclonal HIV-1 antibodies showed that the antigenicity of the native-like Env trimers is largely preserved upon conjugation (Figure 9). Physico-chemical characterisation of the liposomes before and after conjugation suggests that the physical integrity of the liposomes is preserved (Figure S9A,B). Peptide recovery of the encapsulated model T helper peptide OVA 323–339 was around 55% and 95% for UFO Env-liposomes conjugates and SOSIP Env-liposome conjugates, respectively (Figure S9C). Importantly, the recovery of the clinically more relevant T helper cell peptide HBV 2/3 (AGFFLLTRILTIPQSLDSWWTSLN) was similarly high (data not shown). Interestingly, reactions with EDC-crosslinked versions of UFO Env and SOSIP Env did not result in any substantial formation of Env-liposome conjugates (Figure 8). For now, the simplest explanation for this observation is that the reactive/relevant primary amines of the Env trimers have already formed covalent bonds with neighbouring carboxylic acid (i.e., cross-links) and thus are no longer available to engage in the formation of Env-liposome conjugates (personal communication with Quentin Sattentau). Taken together, the results indicate that tag-free conjugation onto cationic liposomes using EDC/Sulfo-NHS chemistry is applicable to UFO and SOSIP constructs, two representative state-of-the-art HIV-1 Env trimer constructs. Moreover, the data show that both, the physical integrity of the liposomes as judged by dynamic light scattering analysis and the antigenicity of the liposome-displayed Env trimers is largely preserved.

## 4. Conclusions

Currently, available protocols for the preparation of covalent Env-liposome conjugates have enabled the straightforward, small-scale preparation of liposomes that display a high number of oriented native-like Env trimers on their surface. Unfortunately, all of these protocols require the use of Env trimers that carry a tag or a specific functional group (e.g., a polyhistidine-tag or a polyhistidine-containing peptide linker and a C-terminal cysteine). This is why we decided to explore EDC/Sulfo-NHS-based chemistry as an alternative approach. The present study systematically addressed the question of what is required to covalently conjugate tag-free, non-functionalised native-like Env trimers onto the surface of carboxyl-functionalised liposomes. Our investigations clearly indicated electrostatic pre-concentration to be of particular importance for the conjugation reaction to proceed. Since the employed native-like Env trimers exhibit a strong negative net charge, successful covalent conjugation was only possible with positively charged liposomes under low-ionic-strength conditions. The preservation of the physical integrity of the liposomes was found to be a function of the Env trimer:liposome ratio and the ionic strength at conjugation. Both are determining factors that severely limited the maximum number of Env trimers that can be coupled onto liposomes. The conformational integrity and antigenicity of the liposome-displayed Env trimers was largely retained by applying a potentially scalable two-step protocol based on the controlled use of β-mercaptoethanol. Conjugation of tag-free, non-functionalised native-like Env trimers onto the surface of liposomes is desirable for many reasons, but as the results of our investigations show, rather difficult to realise.

The main objections or challenges are that it would require the use of positively charged liposomes and that there is only limited control over the number and orientation of the displayed Env trimers. It has been repeatedly reported that the charge of liposome-based vaccines determines their fate (i.e., retention at the point of injection, cellular uptake, and lymph-node trafficking) and the type of immune response they may elicit [80]. The objectives of particulate display of Env trimers are, inter alia, increased lymph-node trafficking and enhanced B cell engagement [2,4,5,6,7,8,9]. Although several studies have shown that positively charged liposomes can indeed be delivered to lymph nodes, further research is required to assess whether their use within the context of nanoparticle-based HIV-1 vaccines is indicated/useful at all [81,82]. Recent studies on the role of nanoscale antigen organisation on the activation of B cells suggest B-cell signalling to be maximised by as few as five antigens rigidly displayed on the surface of 40-nm nanoparticles [79]. The low number of Env trimers per liposome achieved by our tag-free EDC/Sulfo-NHS-based conjugation approach may thus be very well sufficient for an enhanced activation of Env-specific/Env-reactive B cells by B-cell receptor crosslinking (BCR crosslinking). Moreover, the high molar ratio of incorporated T helper cell peptides to surface-displayed Env trimers may be well suited to improve/modulate Env antibody responses by intrastructural help [65,68]. Investigating the immunogenicity and the potential of the prepared Env-liposome conjugates (T helper liposomes) to shape the immune response against the surface-displayed Env trimers is of particular importance but was beyond the scope of the present methodological paper.

With regard to the oriented display, homology models and calculations/estimations of the degree of deprotonation of the Env trimer’s primary amines, suggest that not all primary amines are equally accessible and nucleophilic/reactive (unpublished observations). In concordance with this, recently published homology models of two SOSIP trimers indicate that most lysine residues at the apex of the Env trimer are shielded by glycans [32]. However, many lysine residues on the glycan-free base appear accessible. Ringe et al. found that tag-free, non-functionalised Env trimers coupled to iron oxide nanoparticles using EDC/Sulfo-NHS, induce reduced anti-base non-nAb responses when compared to soluble Env trimers [32]. Based on these observations, they concluded that the Env trimers must have been displayed in a (partly) oriented fashion that occludes the immunodistractive base. In light of these intriguing findings, the use of carboxyl-functionalised lipids other than the PEGylated one we have employed in the present study (i.e., DSPE-PEG14-COOH) seems worth exploring. Carboxyl-functionalised lipids without or with very short spacers such as Succinyl PE, Glutaryl PE or Dodecanyl PE might represent viable candidates for conjugating Env trimers in a fashion that renders their immunodistractive base inaccessible.

## Supplementary Materials

The following are available online at https://www.mdpi.com/1999-4923/12/10/979/s1, Table S1: Buffers I, Table S2: Buffers II, Table S3: Buffers III, Table S4: Buffers IV, Figure S1: Conjugation efficiency and PGT145 reactivity of liposome-displayed UFO Env trimers as a function of the molar excess of EDC used for the activation of cationic, carboxyl-functionalised liposomes, Figure S2: Estimated average number of Env trimers per liposome, Figure S3: Effect of the addition of charged lipids on the physico-chemical properties of carboxyl-functionalised liposomes and the encapsulation of hydrophilic T helper cell peptides, Figure S4: Liposome integrity after the addition of UFO Env trimers as a function of ionic strength, pH and the activation state of the liposomes I (Zeta potential), Figure S5: Liposome integrity after the addition of UFO Env trimers as a function of ionic strength, pH and the activation state of the liposomes II (Diameter and *PdI*), Figure S6: pH-dependent conjugation of UFO Env trimers onto cationic liposomes: physico-chemical characterisation of liposomes before and after conjugation, Figure S7: Conjugation of UFO Env trimers onto cationic liposomes as a function of the molar Env:funct. lipid ratio, Figure S8: Conjugation of UFO Env trimers onto cationic liposomes as a function of the bulk reactant concentration, Figure S9. Conjugation of next-generation HIV-1 immunogens onto cationic, T helper cell peptide-loaded liposomes: peptide recovery and physico-chemical characterisation of liposomes before and after conjugation.
