# Supplementary material for: Conjugation of Native-Like HIV-1 Envelope Trimers onto Liposomes Using EDC/Sulfo-NHS Chemistry: Requirements and Limitations
Source: Pharmaceutics. 2020 Oct 16;12(10):979. doi: 10.3390/pharmaceutics12100979 (PMC7589475; doi:10.3390/pharmaceutics12100979)
Supplement: Supplementary file 1 [file pharmaceutics-12-00979-s001.pdf]

pharmaceutics

MDPI

Supplementary Materials: Conjugation of native-like HIV-1 envelope trimers onto liposomes using EDC/Sulfo-NHS chemistry: requirements and limitations

Ehsan Suleiman, Julia Mayer, Elisabeth Lehner, Bianca Kohlhauser, Alexandra Katholnig, Mirjam Batzoni, Dominik Damm, Vladimir Temchura, Andreas Wagner, Klaus Überla and Karola Vorauer-Uhl

Table 1. Buffers I.

Buffer	CH_3COOH [mM]	$Na(CH_3COO)$ [mM]	NaCl [mM]	Sucrose [mM]	pH adjustment
5 mM ABS pH 4.0	4.1	0.9	150	-	-
5 mM AB sucrose pH 4.0	4.1	0.9	-	300	-
5 mM ABS pH 5.5	4.1	0.9	150	-	w/ NaOH
5 mM AB sucrose pH 5.5	4.1	0.9	-	300	w/ NaOH

Table 2. Buffers II.

Buffer.	$Na_2HPO_4{\cdot}2H_2O$ [mM]	KH_2PO_4 [mM]	NaCl [mM]	Sucrose [mM]
5 mM PBS pH 6.0	0.4	4.6	150	-
5 mM PB sucrose pH 6.0	0.4	4.6	-	300
5 mM PBS pH 6.5	1.5	3.5	150	-
5 mM PB sucrose pH 6.5	1.5	3.5	-	300
5 mM PBS pH 7.5	4.3	0.7	150	-
5 mM PB sucrose pH 7.5	4.3	0.7	-	300

Table 3. Buffers III.

Buffer	$Na_2B_4O_7 \cdot 10\ H_2O$ [mM]	H_3BO_3 [mM]	NaCl [mM]	Sucrose [mM]
5 mM BBS pH 8.5	1.0	4.0	150	-
5 mM BB sucrose pH 8.5	1.0	4.0	-	300

Table 4. Buffers IV.

Buffer	MES [mM]	NaCl [mM]	Sucrose [mM]	pH adjustment
50 mM MBS pH 6.1	50	150	-	w/ NaOH
50 mM MB sucrose pH 6.1	50	-	300	w/ NaOH

pharmaceutics

MDPI

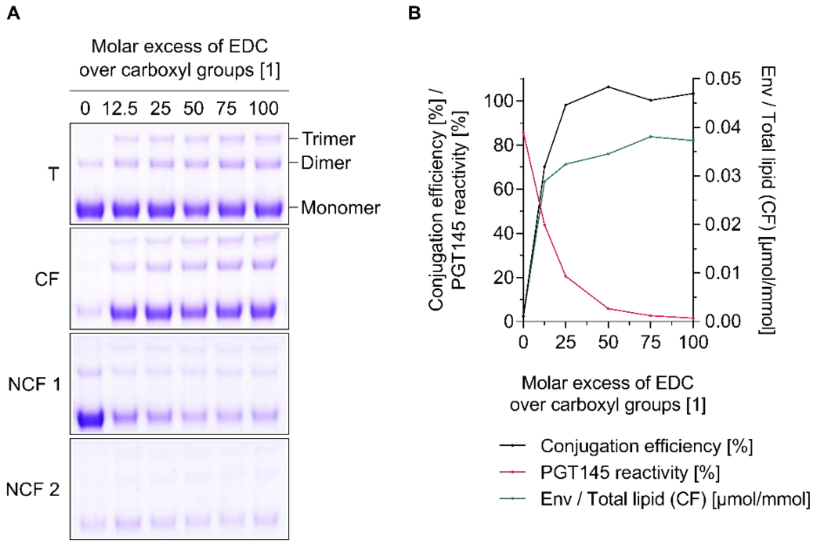

Figure 1. Conjugation efficiency and PGT145 reactivity of liposome-displayed UFO Env trimers as a function of the molar excess of EDC used for the activation of cationic, carboxyl-functionalized liposomes. A. Reducing SDS-PAGE showing the extent of covalent conjugation. B. Conjugation efficiency, PGT145 reactivity and the amount of conjugated UFO Env per total lipid. Data (ELISA and lipid quantification) represent the mean from two analytical replicates. A-B. Liposomes were activated with increasing concentrations of EDC. The ratio of Sulfo-NHS over EDC was kept constant at 1:2.5, e.g., 2 mM Sulfo-NHS and 5 mM EDC. Activations were performed in 50 mM MB sucrose pH 6.1. Excess activation reagents were removed by a single gel filtration step. Liposomes were eluted with 5 mM PB pH 6.0 w/ 15 mM NaCl and w/ 270 mM sucrose. The panel shows the results from a single explorative screening experiment. Abbreviations: T, total conjugation reaction mix; CF, conjugate fraction; NCF 1, non-conjugate fraction 1; NCF 2, non-conjugate fraction 2. See *Materials and Methods (2.8. Ultracentrifugation)* for further information.

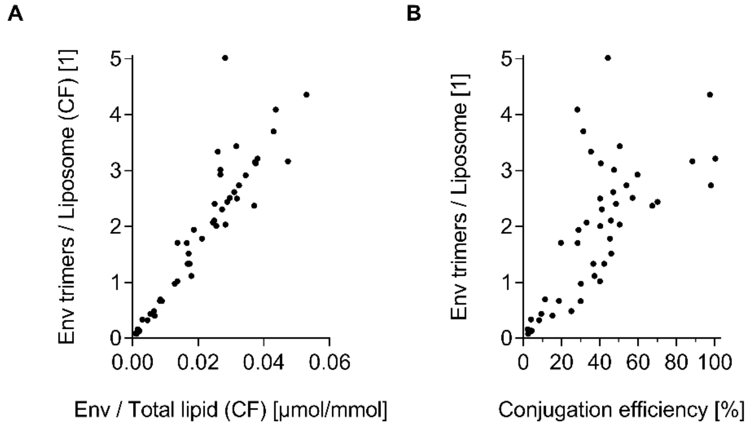

Figure S2. Estimated average number of Env trimers per liposome. A. Relation between the estimated average number of Env trimers per liposome and the molar ratio of Env over total lipid. Total lipid concentrations and Env concentrations were determined as described in the *Methods and Materials* section. The Z-average diameter was used for the estimation. Calculations of the average number of Env trimers per liposome were performed as described elsewhere [1]. **B.** Relation between the estimated average number of Env trimers per liposome and conjugation efficiency of the respective preparation. The panel includes data from all conjugation experiments performed as part of this study.

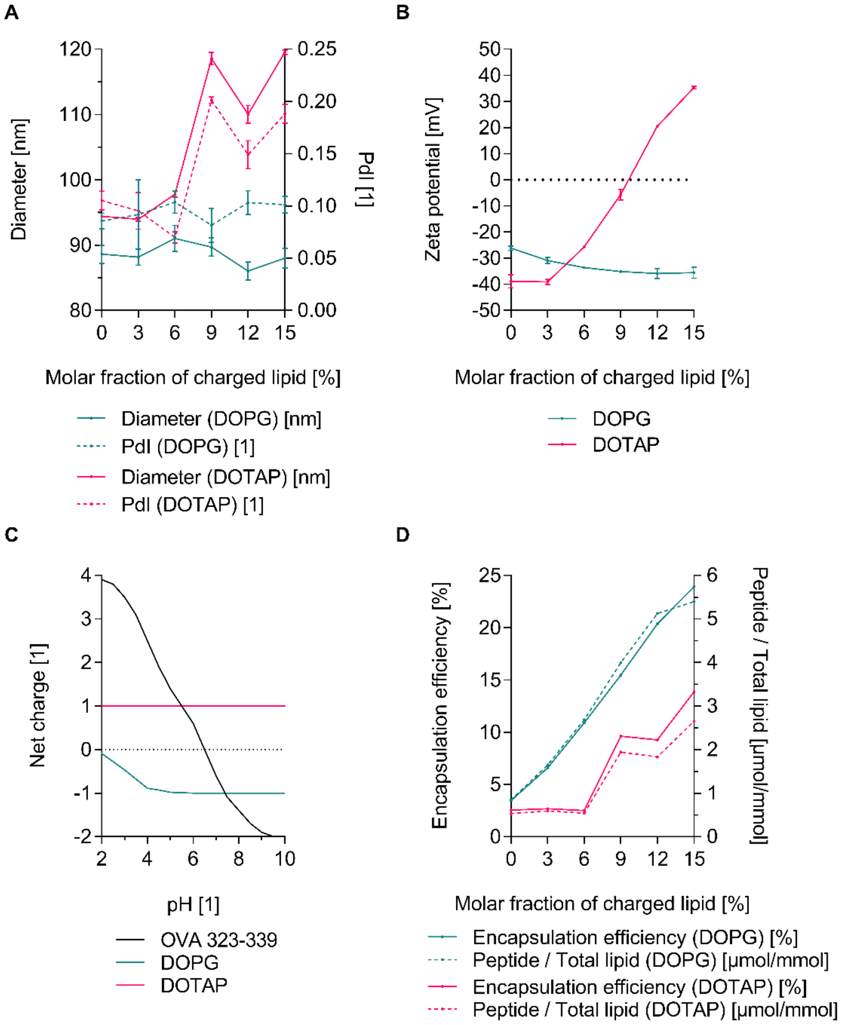

Figure S3. Effect of the addition of charged lipids on the physico-chemical properties of carboxyl-functionalized liposomes and the encapsulation of hydrophilic T helper cell peptides. A-B. (A) Diameter, (i.e., Z-average diameter), PdI and (B) zeta potential of carboxyl-functionalized liposomes with increasing fractions of charged lipid, i.e., DOPG or DOTAP. Data represent the mean ± standard deviation from three analytical replicates. C. Net charge/pH profiles of OVA 323-339 and study-relevant charged lipids. The net charge of OVA 323-339 as a function of pH as calculated by PROTEIN CALCULATOR v3.4 is shown [2]. Data for the DOPG net charge/pH profile were adapted from [3]. DOTAP exhibits a pH-independent net charge of +1 [4]. D. Encapsulation efficiency and the amount of OVA 323-339 per lipid. Data (peptide and lipid quantification) represent the mean from two analytical replicates. The panel shows the results from a single explorative screening experiment.

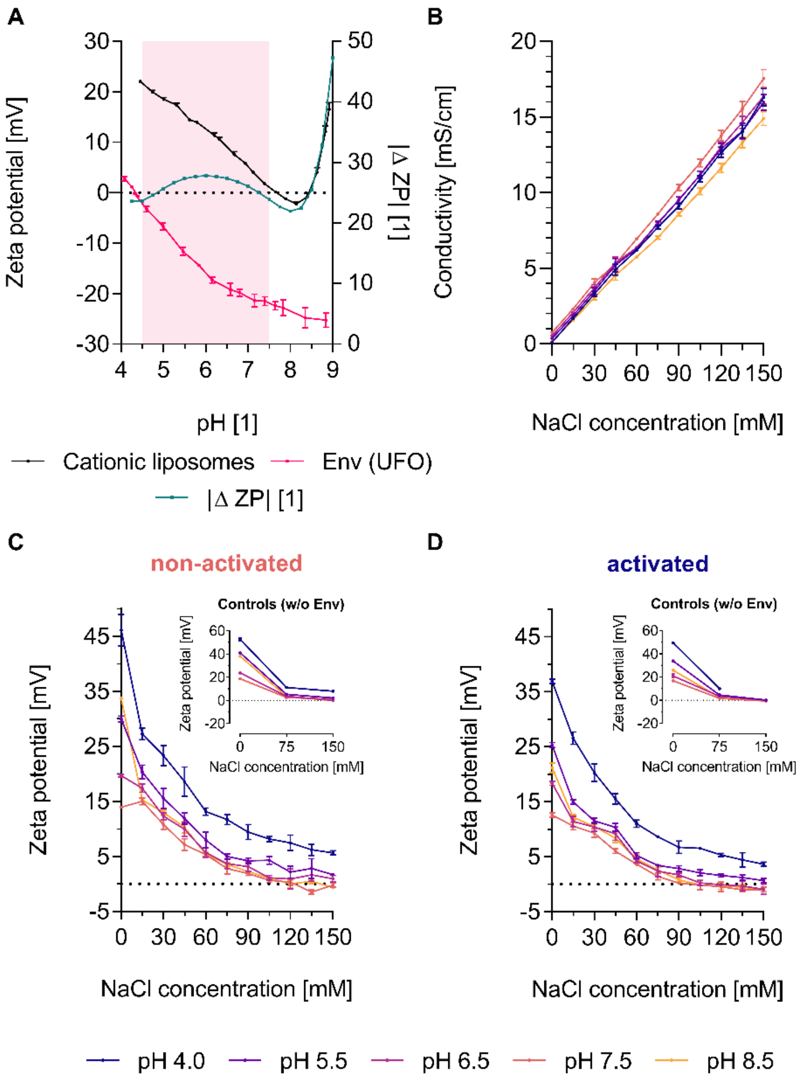

Figure 4. Liposome integrity after the addition of UFO Env trimers as a function of ionic strength, pH and the activation state of the liposomes I (Zeta potential). A. Zeta potential of non-activated, cationic, carboxyl-functionalized liposomes (black line), UFO Env trimers (pink line) and |Δ ZP| (absolute difference in zeta potential between liposomes and Env trimers; blue green line) at low ionic strength (≤ 2 mS/cm) as a function of pH. The light pink area between pH 4.5 to 7.5 indicates the range over which the peptide bond formation (i.e., conjugation) is reported to occur effectively [5]. B. Conductivities (of the buffers) measured during the determination of the zeta potentials shown in Figure S4C and Figure S4D. C-D. Zeta potential of (C) non-activated and (D) activated cationic, carboxyl-functionalized liposomes after the addition of UFO Env trimers. Data represent the mean ± standard deviation from three analytical replicates. The panel shows the results from a single explorative screening experiment.

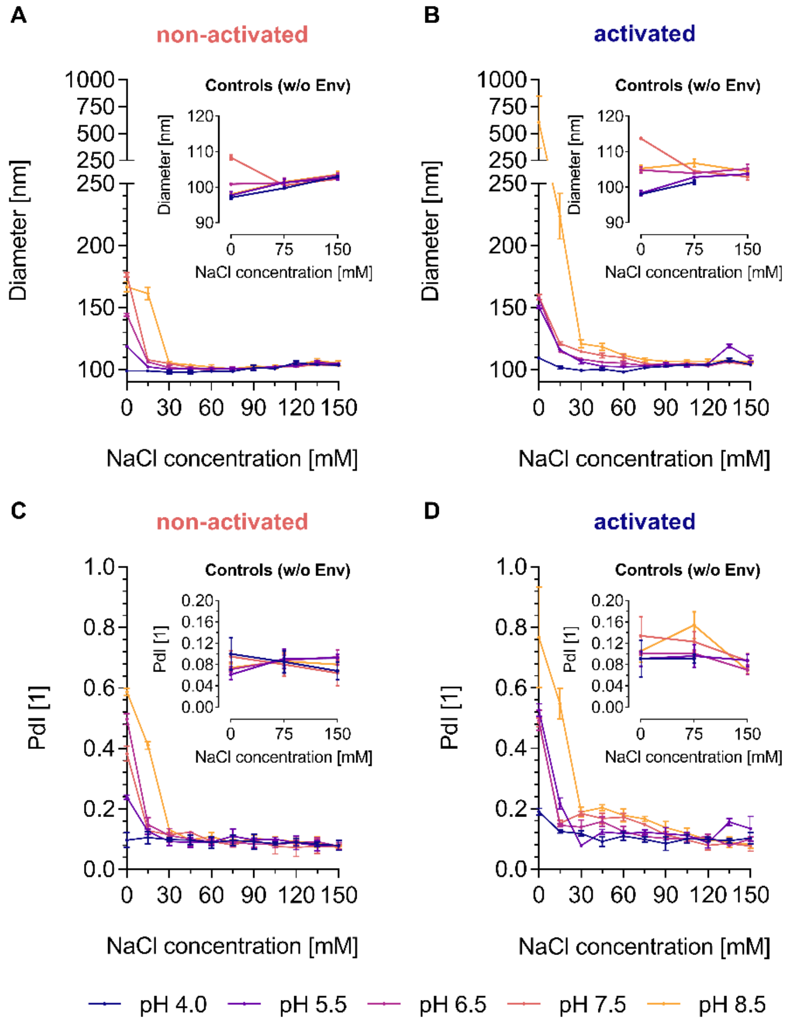

Figure 5. Liposome integrity after the addition of UFO Env trimers as a function of ionic strength, pH and the activation state of the liposomes II (Diameter and PdI). A-B. Diameter, (i.e., Z-average diameter) of (A) non-activated and (B) activated cationic, carboxyl-functionalized liposomes after the addition of UFO Env trimers. C-D. PdI of (C) non-activated and (D) activated cationic, carboxyl-functionalized liposomes after the addition of UFO Env trimers. A-D. Activations were performed in 50 mM MB sucrose pH 6.1. Excess activation reagents were removed by a single gel filtration step. One half of the volume of the activated liposomes was eluted with low-ionic strength buffer of desired pH (e.g., 5 mM AB pH 4.0 w/ 300 mM sucrose) while the other half was eluted with high-ionic strength buffer with the same pH (e.g., 5 mM AB pH 4.0 w/ 150 mM NaCl). Liposomes were then immediately mixed to give the sodium chloride concentration required for the experiment. The different pH values at which the experiment was performed were realized by eluting the activated liposomes with a range of different buffers (5 mM AB pH 4.0, 5 mM AB pH 5.5, 5 mM PB pH 6.5, 5 mM PB pH 7.5 and 5 mM BB pH 8.5) which were all prepared as low-ionic strength and high-ionic strength version. Data represent the mean ± standard deviation from three analytical replicates. The panel shows the results from a single explorative screening experiment.

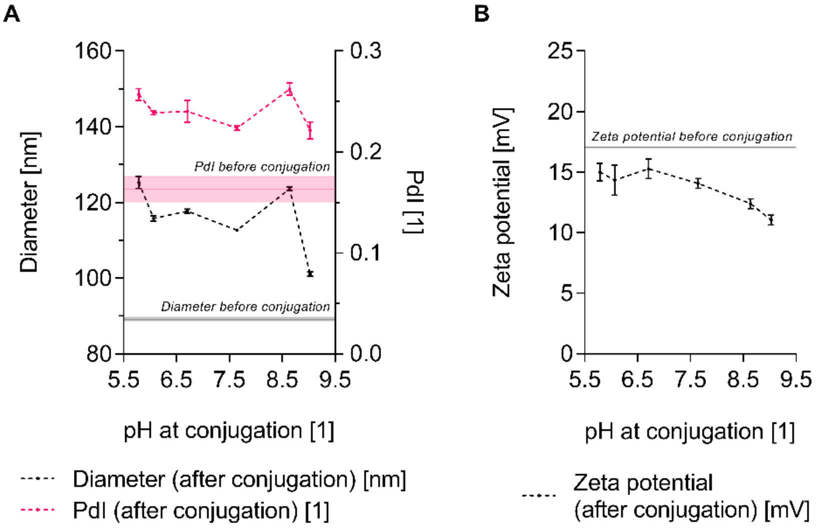

Figure 6. pH-dependent conjugation of UFO Env trimers onto cationic liposomes: physico-chemical characterization of liposomes before and after conjugation. A-B. (A) Diameter, (i.e., Z-average diameter), PdI and (B) zeta potential before and after conjugation. Data (the points and the horizontal lines indicate diameter and PdI before conjugation) represent the mean ± standard deviation from three analytical replicates. The panel shows the results from a single explorative screening experiment.

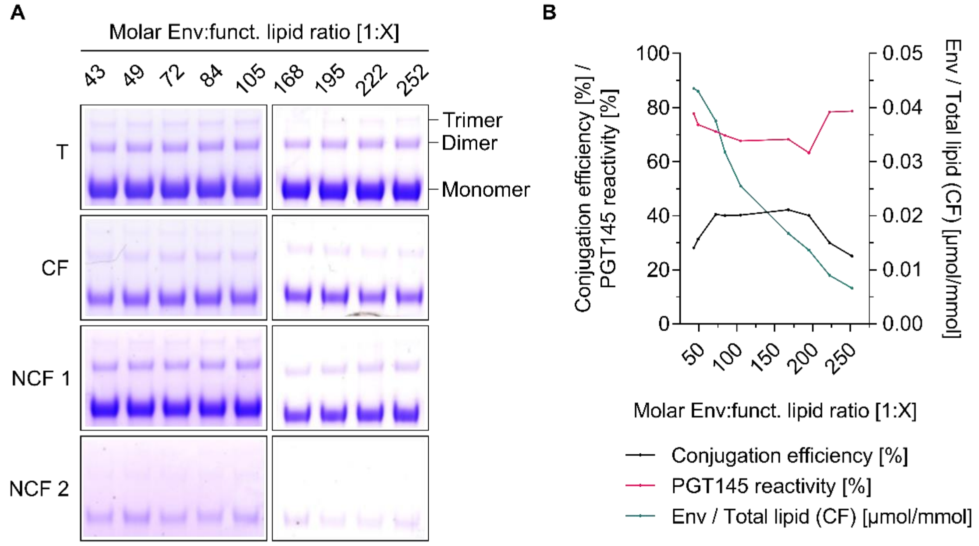

Figure 7. Conjugation of UFO Env trimers onto cationic liposomes as a function of the molar Env:funct. lipid ratio. A. Reducing SDS-PAGE showing the extent of covalent conjugation as a function of the molar Env:funct. lipid ratio during conjugation, i.e., the molar ratio of the N-termini of the trimeric envelope protein Env to the accessible carboxyl groups on the outer surface of the carboxyl-functionalized liposome. **B.** Conjugation efficiency, PGT145 reactivity and the amount of conjugated UFO Env per total lipid. Data (ELISA and lipid quantification) represent the mean from two analytical replicates. **A-B.** Liposomes were activated in 5 mM PB pH 6.0 w/ 15 mM NaCl and w/ 270 mM sucrose. Excess EDC was chemically deactivated by the addition of β-ME. Conjugation reactions were performed at a constant nominal Env trimer concentration of 706 nM (254 µg/mL) and varying nominal total lipid concentrations in the range of 3.0 mM to 18.5 mM. The panel shows the results from a single explorative screening experiment. **Abbreviations:** T, total conjugation reaction

mix; CF, conjugate fraction; NCF 1, non-conjugate fraction 1; NCF 2, non-conjugate fraction 2. See *Materials and Methods (2.8. Ultracentrifugation)* for further information.

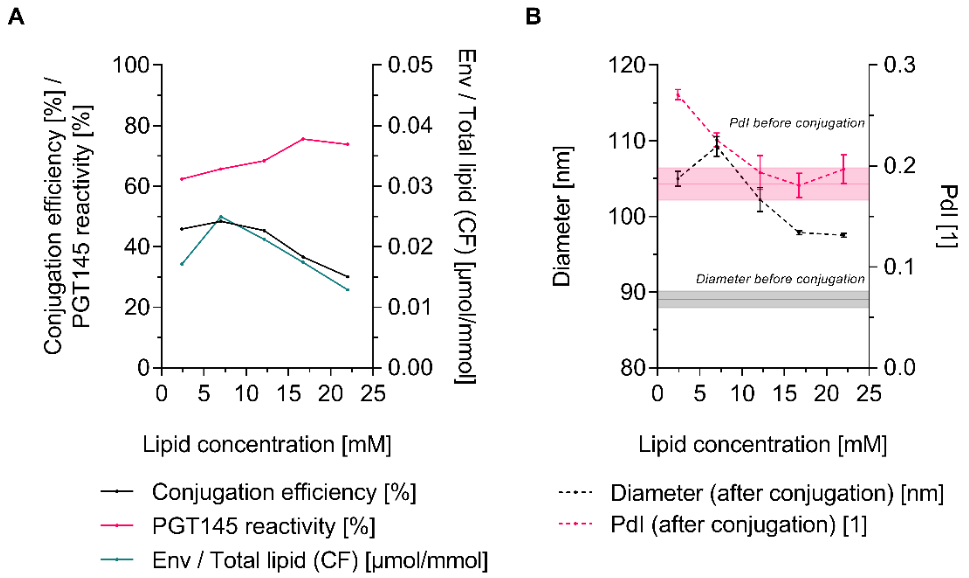

Figure S8. Conjugation of UFO Env trimers onto cationic liposomes as a function of the bulk reactant concentration. A. Conjugation efficiency, PGT145 reactivity and the amount of conjugated UFO Env per total lipid as a function of the bulk reactant concentration. The molar Env:funct. lipid ratio during conjugation, (i.e., the molar ratio of the N-termini of the trimeric envelope protein Env to the accessible carboxyl groups on the outer surface of the carboxyl-functionalized liposome) was kept constant at 1:94. The lipid concentration, (i.e., total lipid concentration during conjugation) is used to represent the equally increasing concentrations of lipid and Env trimers used for conjugation. Data (ELISA and lipid quantification) represent the mean from two analytical replicates. **B.** Diameter, (i.e., Z-average diameter) and PdI before and after conjugation. Data (the points and the horizontal lines indicate diameter and PdI before conjugation) represent the mean ± standard deviation from three analytical replicates. **A-B.** Liposomes were activated in 5 mM PB pH 6.0 w/ 15 mM NaCl and w/ 270 mM sucrose. Excess EDC was chemically deactivated by the addition of β-ME. The panel shows the results from a single explorative screening experiment.

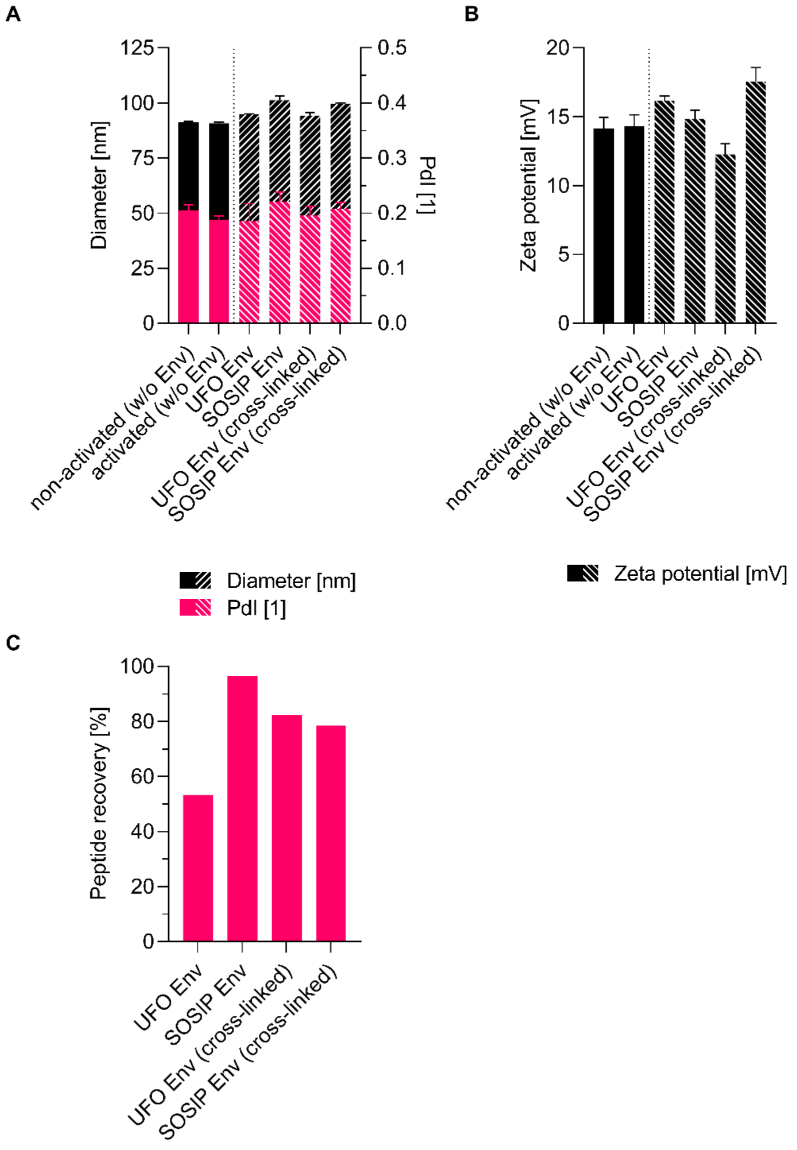

Figure 9. Conjugation of next-generation HIV-1 immunogens onto cationic, T helper cell peptide-loaded liposomes: peptide recovery and physico-chemical characterization of liposomes before and after conjugation. A-B. (A) Diameter, (i.e., Z-average diameter), PdI and (B) zeta potential before and after conjugation. Data (the hatched bars that represent the liposomes' state after conjugation as well as the full bars that represent the liposomes' state before conjugation) represent the mean ± standard deviation from three analytical replicates. C. Peptide recovery in the *conjugate fractions* (CF), i.e., Env-liposome conjugates without non-conjugated Env and without free (non-encapsulated) peptide. The peptide recovery was calculated on the basis of the peptide and lipid concentration of the liposomes before conjugation/activation and that of the *conjugate fraction.* Calculations were performed taking into account the centrifugation-related lipid losses. Data (peptide quantification, lipid quantification and the recovery calculated on the basis of these) represent the mean from two analytical replicates. The panel shows the results from a single explorative screening experiment.

References

1. Steichen, J.M.; Kulp, D.W.; Tokatlian, T.; Escolano, A.; Dosenovic, P.; Stanfield, R.L.; McCoy, L.E.; Ozorowski, G.; Hu, X.; Kalyuzhniy, O.; et al. HIV Vaccine Design to Target Germline Precursors of Glycan-Dependent Broadly Neutralizing Antibodies. *Immunity* **2016**, *45*, 483–496, doi:10.1016/j.immuni.2016.08.016.
2. Protein Calculator v3.4 Available online: http://protcalc.sourceforge.net/ (accessed on May 28, 2019).
3. Marsh, D. *Handbook of Lipid Bilayers*; 2nd ed.; CRC Press, 2013; ISBN 978-1-4200-8833-5.

4. Simberg, D.; Weisman, S.; Talmon, Y.; Barenholz, Y. DOTAP (and other cationic lipids): chemistry, biophysics, and transfection. *Crit. Rev. Ther. Drug Carrier Syst.* **2004**, *21*, 257–317, doi:10.1615/CritRevTherDrugCarrierSyst.v21.i4.10.
5. Hermanson, G.T. Zero-Length Crosslinkers. In *Bioconjugate Techniques*; Academic Press (Elsevier Inc.), 2013; pp. 259–273 ISBN 978-0-12-382239-0.
